# Prognostic value of programmed death-ligand 1 status in Japanese patients with renal cell carcinoma

**DOI:** 10.1007/s10147-021-01993-x

**Published:** 2021-07-21

**Authors:** Motohide Uemura, Noboru Nakaigawa, Naoto Sassa, Katsunori Tatsugami, Kenichi Harada, Toshinari Yamasaki, Nobuaki Matsubara, Takuya Yoshimoto, Yuki Nakagawa, Tamaki Fukuyama, Mototsugu Oya, Nobuo Shinohara, Hirotsugu Uemura, Toyonori Tsuzuki

**Affiliations:** 1 Department of Urology, Osaka University Graduate School of Medicine, 2-2 Yamadaoka, Suita, Osaka 565-0871 Japan; 2 Department of Urology, Yokohama City University Graduate School of Medicine, 22-2 Seto, Kanazawa Ward, Yokohama, Kanagawa 236-0027 Japan; 3 Department of Urology, Nagoya University Graduate School of Medicine, Furocho, Chikusa Ward, Nagoya, Aichi 464-8601 Japan; 4 Department of Urology, Kyushu University Graduate School of Medical Sciences, 3-1-1, Maidashi, Higashi-ku, Fukuoka City, 812-8582 Japan; 5 Division of Urology, Department of Surgery Related, Kobe University Graduate School of Medicine, 7-5-2, Kusunoki-cho, Chuo-ku, Kobe, Hyogo 650-0017 Japan; 6 Department of Urology, Kyoto University Graduate School of Medicine, Yoshidakonoecho, Sakyo Ward, Kyoto, 606-8501 Japan; 7 Department of Breast and Medical Oncology, National Cancer Center Hospital East, 6-5-1 Kashiwanoha, Kashiwa, Chiba 277-8577 Japan; 8 Biometrics Department, Chugai Pharmaceutical Co., Ltd., Nihonbashi Muromachi 2-1-1, Chuo City, Tokyo 103-8324 Japan; 9 Medical Affairs Division, Chugai Pharmaceutical Co., Ltd., Nihonbashi Muromachi 2-1-1, Chuo City, Tokyo 103-8324 Japan; 10 Department of Urology, Keio University School of Medicine, 35 Shinanomachi, Shinjuku City, Tokyo 160-8582 Japan; 11 Department of Renal and Genitourinary Surgery, Hokkaido University Graduate School of Medicine, Kita 15, Nishi 7, Kita-ku, Sapporo, 060-8638 Japan; 12 Department of Urology, Kindai University, Faculty of Medicine, 377‐2 Ohnohigashi, Osaka-Sayama City, Osaka 589‐8511 Japan; 13 Department of Surgical Pathology, Aichi Medical University Hospital, 1-1 Yazakokarimata, Nagakute, Aichi 480-1195 Japan; 14 Present Address: Department of Urology, Aichi Medical University Hospital, 1-1 Yazakokarimata, Nagakute, Aichi 480-1195 Japan; 15 Present Address: Department of Urology, Kitakyushu Municipal Medical Center, 2-1-1 Bashaku, Kokurakita Ward, Kitakyushu, Fukuoka 802-0077 Japan

**Keywords:** Japan, PD-L1, Prognosis, Renal cell carcinoma, Recurrence

## Abstract

**Background:**

Programmed death-ligand 1 (PD-L1) positivity is associated with poor prognosis in renal cell carcinoma (RCC). Because the prognostic impact and effect of confounding factors are less known, we investigated the prognostic significance of PD-L1 expression in Japanese patients with recurrent/metastatic RCC who started systemic therapy in 2010–2015.

**Methods:**

This multicenter, retrospective study recruited patients from 29 Japanese study sites who had prior systemic therapy for RCC (November 2018 to April 2019) and stored formalin-fixed paraffin-embedded primary lesion samples. The primary outcome was overall survival (OS) by PD-L1 expression. Secondary outcomes included OS in subgroups and duration of first- and second-line therapies by PD-L1 expression. OS distributions were estimated using Kaplan–Meier methodology.

**Results:**

PD-L1 expression (on immune cells [IC] ≥ 1%) was observed in 315/770 (40.9%) patients. PD-L1 positivity was more prevalent in patients with poor risk per both Memorial Sloan Kettering Cancer Center [MSKCC] and International Metastatic RCC Database Consortium, and high-risk pathological features (higher clinical stage, nuclear grade and sarcomatoid features). Median OS for PD-L1–positive patients was 30.9 months (95% CI 25.5–35.7) versus 37.5 months (95% CI 34.0–42.6) for PD-L1–negative patients (HR 1.04 [90% CI 0.89–1.22, *p* = 0.65]; stratified by MSKCC risk and liver metastases). Propensity score weight (PSW)-adjusted OS was similar between PD-L1–positive and –negative patients (median 34.4 versus 31.5 months; estimated PSW-adjusted HR 0.986).

**Conclusions:**

This study suggests PD-L1 status was not an independent prognostic factor in recurrent/metastatic RCC during the study period because PD-L1 positivity was associated with poor prognostic factors, especially MSKCC risk status.

**Supplementary Information:**

The online version contains supplementary material available at 10.1007/s10147-021-01993-x.

## Introduction

Targeted therapies (e.g., vascular endothelial growth factor receptor [VEGFR] tyrosine kinase inhibitors [TKIs] and mammalian target of rapamycin [mTOR] inhibitors) have been the standard of care for renal cell carcinoma (RCC) since 2008 in Japan [1]. Immunotherapies targeting the programmed death 1/programmed death-ligand 1 (PD-[L]1) pathway and anti–cytotoxic T-lymphocyte–associated antigen 4 (CTLA-4) recently remodeled the metastatic RCC (mRCC) treatment landscape. Checkpoint inhibitors (CPIs) have demonstrated promising efficacies in the first-line (1L) setting in combination with anti–CTLA-4 or VEGFR TKIs [2–4].

PD-L1 is an established biomarker for antitumor T-cell response in other cancers but not in mRCC [5]. Although better outcomes were noted in PD-L1–positive populations, anti-tumor effects were also observed in PD-L1–negative populations compared with controls[2–4]. Thus, routine PD-L1 testing is not standard for treating mRCC with CPIs. The prognostic role of PD-L1 expression in RCC has been investigated in several studies. High PD-L1 expression (on tumor cells [TC] or tumor-infiltrating immune cells [IC]) correlated with shorter overall survival (OS) and worse prognostic features (high nuclear grade, sarcomatoid differentiation, higher T stage, and poor International Metastatic RCC Database Consortium [IMDC] risk status) [6–9]. Hence, PD-L1 expression was recently recognized as a negative prognostic factor in RCC [10].

Several patient characteristics can affect clinical outcomes in RCC. Two-year survival rates (per IMDC risk classification) were unsatisfactory for patients with poor risk mRCC (7% versus 75% and 53% [favorable and intermediate]) [11]. Poor outcomes have also been reported for patients with mRCC and sarcomatoid components [12]. Tumor immune phenotype is another potential prognostic biomarker; prognosis for clear cell RCC worsens with cluster of differentiation 8–positive (CD8 +) T-cell infiltration into tumor tissue and checkpoint molecule expression [13].

PD-L1 expression may not only be a predictive biomarker for CPI treatment, but also a negative prognostic marker in RCC. Thus, clarifying the mechanism behind its potential prognostic significance is important in understanding RCC biology. In this multicenter, retrospective study, we investigated the prognostic significance of PD-L1 expression on OS in a large cohort of Japanese patients treated with systemic therapy for recurrent/metastatic RCC before CPIs became the standard of care for 1L treatment. We also explored whether PD-L1 would remain an independent prognostic factor after adjusting for baseline characteristics, which would increase its clinical significance.

## Materials and methods

### Patients

Samples were collected between November 2018 and June 2019 from 29 Japanese study sites that provided guideline-based, standard-of-care treatment. Patients aged ≥ 20 years underwent nephrectomy, had stored formalin-fixed paraffin-embedded (FFPE) surgical samples of the primary lesion, and started systemic therapy for recurrent/metastatic RCC between January 2010 and December 2015. Since long-term storage of paraffin blocks generally does not significantly impact immunohistochemistry, there was no limitation on the sample collection time of FFPE surgical samples [14]. Exclusion criteria were coexisting malignancies (from nephrectomy to death) or first-line (1L) treatment with anti–CTLA-4/anti–PD-(L)1 for recurrent/metastatic RCC. Patients with tumor tissue samples were recruited and informed consent was obtained. This study is registered under UMIN (UMIN000034131) and was performed after approval by each institutional review board (IRB) of the 29 study sites. Approval from the IRB of MINS, a non-profit organization, was also obtained.

More details on patients can be found in the supplementary methods in the Online Resource.

### Study design and outcomes

This multicenter, retrospective study compared OS by PD-L1 expression status to determine its prognostic effect. The primary outcome was OS, which is defined as the time from initiation of 1L therapy to death from any cause, according to PD-L1 status (positive versus negative). Secondary outcomes included OS in subgroups by PD-L1 status, expression level (four IC levels), and duration of 1L and second-line (2L) therapy by PD-L1 status.

### Pathology and immunohistochemistry

Representative FFPE samples were selected by pathologists in each institution and evaluated by a central pathologist. Samples had to be of good quality and had to have an adequate number of tumor cells for PD-L1 evaluation. Pathological assessments were conducted to determine the histologic type such as Fuhrman grade (1–4), World Health Organization (WHO)/International Society of Urologic Pathologists (ISUP) grade (1–4), tumor necrosis, lymphovascular infiltration, sarcomatoid component, and growth pattern [15, 16].

All samples were sent to the central laboratory (SRL, Inc.) for hematoxylin and eosin staining and immunohistochemistry staining using standardized protocols. PD-L1 expression was evaluated independently by two central pathologists by immunohistochemistry using the VENTANA SP142 assay (Ventana Medical Systems, Inc., 740–4859). Based on IC expression levels, patients were classified as either PD-L1 negative (IC0; IC < 1%), or PD-L1 positive (IC1 [IC: ≥ 1% but < 5%], IC2 [IC: ≥ 5% but < 10%] or IC3 [IC ≥ 10%]).

Immune phenotype assessment was performed using CD8 immunostaining [17]. Based on three characterizations of T-cell activity from tumor biopsies, patients were categorized into immune-desert (T-cell absence), immune-excluded (T-cell accumulation but not in tumor core), and immune-inflamed (T-cells infiltration but not functioning) phenotypes [18, 19].

Details on pathology and immunohistochemistry can be found in the Supplementary methods.

### Statistical analyses

The full analysis set used for this study included all enrolled patients, except those with indeterminate PD-L1 expression. Based on previous reports and clinical significance, a median OS of 26 months for the PD-L1–negative group and 20 months for the PD-L1–positive group was assumed, leading to a hypothesized true HR of 1.3 for this study [4, 6]. When 80% power is guaranteed at a significance level of 10%, two-sided, the required number of events would be 359. To ensure the required number of events, and accounting for specimens with indeterminate PD-L1 status as well as dropouts due to consent withdrawal, enrollment of approximately 600 patients was planned. nQuery Advisor 7.0 (Statistical Solutions Ltd, Ireland) was used to calculate the number of events.

OS distributions were estimated using the Kaplan–Meier (KM) method, and CI of the median was estimated using the Brookmeyer-Crowley technique [20]. The superiority hypothesis was tested with a two-sided significance level of 10% using log-rank test, stratified by MSKCC risk criteria (favorable, intermediate, or poor) and liver metastases. These stratification factors were selected based on expected substantial effects on OS and a previous clinical trial, which evaluated PD-L1 status by SP142, considering the non-randomized nature of this study [2]. Stratified HRs and their 90% CIs were estimated using the stratified Cox regression model (PD-L1 positive: test arm; PD-L1 negative: control arm). Unstratified and subgroup analyses were also performed. In addition, propensity score weight (PSW) analysis was conducted to evaluate the robustness of the primary analysis [21]. A logistic regression model was used to predict the score for each patient, defined as the probability of being PD-L1 positive (explanatory variables in Supplementary Tables S2, S3). After confirming sufficient overlap in propensity score distribution, matching weights method was applied [22].

## Results

### Clinicopathological characteristics by PD-L1 status at initial diagnosis and nephrectomy

Of 777 samples collected, seven samples were not PD-L1–evaluable. Hence, only 770 patients were recruited (PD-L1 positive: 315; PD-L1 negative: 455). Characteristics between groups were generally balanced for sex, histological type, lymphovascular invasion, and growth pattern (Table 1). Fewer patients in the PD-L1–positive group had undergone radical nephrectomy than in the PD-L1–negative group (38.1% versus 57.4%).

**Table 1:** Clinicopathological characteristics at the time of initial diagnosis and nephrectomy and at the time of first-line treatment, by PD-L1 status

Category	Full analysis set		
	PD-L1 positive (IC1/2/3)	PD-L1 negative (IC0)	Total
	*n* = 315	*n* = 455	*N* = 770
At initial diagnosis and nephrectomy			
Sex (male), *n* (%)	235 (74.6)	356 (78.2)	591 (76.8)
Stage at initial diagnosis, *n* (%)			
I	28 (8.9)	84 (18.5)	112 (14.5)
II	17 (5.4)	35 (7.7)	52 (6.8)
III	58 (18.4)	111 (24.4)	169 (21.9)
IV	207 (65.7)	210 (46.2)	417 (54.2)
Unknown	5 (1.6)	15 (3.3)	20 (2.6)
Sample collection year, *n* (%)			
Before 2009	58 (18.4)	135 (29.7)	193 (25.1)
2010–2012	133 (42.2)	207 (45.5)	340 (44.2)
2013–2015	123 (39.0)	112 (24.6)	235 (30.5)
After 2016	1 (0.3)	1 (0.2)	2 (0.3)
Surgery objective, *n* (%)			
Radical nephrectomy	120 (38.1)	261 (57.4)	381 (49.5)
Cytoreductive nephrectomy	195 (61.9)	194 (42.6)	389 (50.5)
Metastatic site at initial diagnosis, *n* (%)^a^			
Lung	134 (42.5)	121 (26.6)	255 (33.1)
Bone	59 (18.7)	58 (12.7)	117 (15.2)
Lymph node	59 (18.7)	45 (9.9)	104 (13.5)
Liver	9 (2.9)	22 (4.8)	31 (4.0)
Adrenal gland	15 (4.8)	19 (4.2)	34 (4.4)
Pleura	12 (3.8)	4 (0.9)	16 (2.1)
Pathological feature by central pathology			
Immune phenotype, *n* (%)			
Excluded	234 (74.3)	144 (31.6)	378 (49.1)
Inflamed	52 (16.5)	7 (1.5)	59 (7.7)
Desert	29 (9.2)	304 (66.8)	333 (43.2)
Histology (central pathology), *n* (%)			
Clear cell RCC	293 (93.0)	403 (88.6)	696 (90.4)
Papillary RCC	7 (2.2)	28 (6.2)	35 (4.5)
Chromophobe RCC	1 (0.3)	6 (1.3)	7 (0.9)
Spindle cell carcinoma	6 (1.9)	1 (0.2)	7 (0.9)
Others	8 (2.5)	17 (3.7)	25 (3.2)
Fuhrman grade, *n* (%)			
1	0 (0.0)	1 (0.2)	1 (0.1)
2	50 (15.9)	203 (44.6)	253 (32.9)
3	176 (55.9)	198 (43.5)	374 (48.6)
4	88 (27.9)	50 (11.0)	138 (17.9)
Indeterminable	1 (0.3)	3 (0.7)	4 (0.5)
WHO/ISUP grade, *n* (%)			
1	0 (0.0)	1 (0.2)	1 (0.1)
2	67 (21.3)	233 (51.2)	300 (39.0)
3	132 (41.9)	152 (33.4)	284 (36.9)
4	115 (36.5)	66 (14.5)	181 (23.5)
Indeterminable	1 (0.3)	3 (0.7)	4 (0.5)
Necrosis, *n* (%)			
Present	187 (59.4)	144 (31.6)	331 (43.0)
Absent	128 (40.6)	309 (67.9)	437 (56.8)
Indeterminable	0 (0.0)	2 (0.4)	2 (0.3)
Lymphovascular invasion, *n* (%)			
Present	93 (29.5)	96 (21.1)	189 (24.5)
Absent	203 (64.4)	336 (73.8)	539 (70.0)
Indeterminable	19 (6.0)	23 (5.1)	42 (5.5)
Sarcomatoid component, *n* (%)			
Present	60 (19.0)	27 (5.9)	87 (11.3)
Absent	255 (81.0)	428 (94.1)	683 (88.7)
Growth pattern, *n* (%)			
Expansive	107 (34.0)	171 (37.6)	278 (36.1)
Infiltrative	85 (27.0)	98 (21.5)	183 (23.8)
Indeterminable	123 (39.0)	186 (40.9)	309 (40.1)
At 1L therapy			
Metastatic site, *n* (%)^a^			
Lung	215 (68.3)	286 (62.9)	501 (65.1)
Bone	78 (24.8)	120 (26.4)	198 (25.7)
Lymph node	83 (26.3)	87 (19.1)	170 (22.1)
Liver	28 (8.9)	51 (11.2)	79 (10.3)
Adrenal gland	19 (6.0)	27 (5.9)	46 (6.0)
Pancreas	13 (4.1)	17 (3.7)	30 (3.9)
Brain	14 (4.4)	15 (3.3)	29 (3.8)
Kidney (recurrent)	11 (3.5)	11 (2.4)	22 (2.9)
Pleura	19 (6.0)	9 (2.0)	28 (3.6)
Age category (years), *n* (%)			
< 40	2 (0.6)	14 (3.1)	16 (2.1)
40 ≤ 50	23 (7.3)	24 (5.3)	47 (6.1)
50 ≤ 60	51 (16.2)	85 (18.7)	136 (17.7)
60 ≤ 70	121 (38.4)	170 (37.4)	291 (37.8)
≥ 70	118 (37.5)	162 (35.6)	280 (36.4)
Liver metastases, *n* (%)			
Present	28 (8.9)	51 (11.2)	79 (10.3)
Absent	286 (90.8)	404 (88.8)	690 (89.6)
Unknown	1 (0.3)	0 (0.0)	1 (0.1)
MSKCC risk, *n* (%)			
Favorable	36 (11.4)	135 (29.7)	171 (22.2)
Intermediate	224 (71.1)	287 (63.1)	511 (66.4)
Poor	55 (17.5)	33 (7.3)	88 (11.4)
IMDC risk, *n* (%)			
Favorable	34 (10.8)	119 (26.2)	153 (19.9)
Intermediate	189 (60.0)	268 (58.9)	457 (59.4)
Poor	92 (29.2)	68 (14.9)	160 (20.8)

*IC0/1/2/3* PD-L1 expression level on tumor-infiltrating immune cells; *IMDC* International Metastatic RCC Database Consortium; *MSKCC* Memorial Sloan Kettering Cancer Center; *PD-L1* programmed death-ligand 1; *RCC* renal cell carcinoma; *WHO/ISUP* World Health Organization/International Society of Urologic Pathologists

^a^Only sites with prevalence ≥ 3% are presented

Immune phenotype (CD8-infiltration type) composition differed between PD-L1–positive (excluded: 74.3%; inflamed: 16.5%; desert: 9.2%) and PD-L1–negative groups (excluded: 31.6%; inflamed: 1.5%; desert: 66.8%; Table 1). Furthermore, PD-L1 positivity was numerically higher in patients with higher clinical stage, higher nuclear grade (WHO/ISUP grade and Fuhrman grade), and sarcomatoid disease. The two groups were imbalanced in sample collection year; PD-L1 positivity was lower in older samples.

### Clinical characteristics by PD-L1 status at the time of 1L therapy

PD-L1 positivity was similar based on age and metastatic site, including liver metastases (Table 1). A higher proportion of PD-L1–positive than –negative patients had poor prognostic features at 1L therapy: poor MSKCC risk, 17.5% versus 7.3%; poor IMDC risk, 29.2% versus 14.9%. Fewer PD-L1–positive patients had the following good prognostic features at 1L therapy: favorable MSKCC risk, 11.4% versus 29.7%; favorable IMDC risk, 10.8% versus 26.2%.

### Systemic therapy for metastatic/recurrent RCC

Sunitinib (VEGFR TKI) was the most common 1L therapy for PD-L1–positive and –negative groups (51.7 and 53.4%; Table 2). Second-line therapies were received by 70.8% of PD-L1–positive and 75.6% of –negative patients. Axitinib (VEGFR TKI) was the most common 2L therapy (28.3 and 34.3%). Third-line therapy was received by 39.0% of PD-L1–positive and 44.8% of –negative patients (Table 2). Everolimus (mTOR inhibitor) was the most common third-line therapy (11.1 and 13.8%), and nivolumab was the only CPI received (5.1 and 7.0%). Fourth-line therapies were received by 15.9% of PD-L1–positive and 23.3% of –negative patients.

**Table 2:** Summary of systemic therapy for metastatic/recurrent RCC

Systemic therapy	Full analysis set		
	PD-L1 positive (IC1/2/3)	PD-L1 negative (IC0)	Total
	*n* = 315	*n* = 455	*N* = 770
1L			
Patients who received 1L therapy, *n* (%)^a^	315 (100.0)	455 (100.0)	770 (100.0)
VEGFR TKI			
Sunitinib	163 (51.7)	243 (53.4)	406 (52.7)
Sorafenib	43 (13.7)	48 (10.5)	91 (11.8)
Axitinib	11 (3.5)	24 (5.3)	35 (4.5)
Pazopanib	10 (3.2)	18 (4.0)	28 (3.6)
mTOR inhibitor			
Temsirolimus	29 (9.2)	21 (4.6)	50 (6.5)
Interferon-alpha	46 (14.6)	84 (18.5)	130 (16.9)
Reasons for discontinuation of 1L therapy, *n* (%)			
Progressive disease	191 (60.6)	265 (58.2)	456 (59.2)
AEs	84 (26.7)	131 (28.8)	215 (27.9)
Others	31 (9.8)	37 (8.1)	68 (8.8)
Unknown	3 (1.0)	5 (1.1)	8 (1.0)
2L			
Patients who received 2L therapy, *n* (%)^a^	223 (70.8)	344 (75.6)	567 (73.6)
VEGFR TKI			
Axitinib	89 (28.3)	156 (34.3)	245 (31.8)
Sunitinib	40 (12.7)	49 (10.8)	89 (11.6)
Sorafenib	14 (4.4)	37 (8.1)	51 (6.6)
mTOR inhibitor			
Everolimus	48 (15.2)	53 (11.6)	101 (13.1)
Temsirolimus	12 (3.8)	18 (4.0)	30 (3.9)
Reasons for discontinuation of 2L therapy, *n* (%)			
Progressive disease	124 (39.4)	205 (45.1)	329 (42.7)
AEs	67 (21.3)	80 (17.6)	147 (19.1)
Others	19 (6.0)	28 (6.2)	47 (6.1)
Unknown	3 (1.0)	4 (0.9)	7 (0.9)
3L			
Patients who received 3L therapy, *n* (%)^a^	123 (39.0)	204 (44.8)	327 (42.5)
VEGFR TKI			
Axitinib	27 (8.6)	40 (8.8)	67 (8.7)
Sunitinib	12 (3.8)	18 (4.0)	30 (3.9)
Pazopanib	10 (3.2)	18 (4.0)	28 (3.6)
Sorafenib	10 (3.2)	16 (3.5)	26 (3.4)
mTOR inhibitor			
Everolimus	35 (11.1)	63 (13.8)	98 (12.7)
Temsirolimus	10 (3.2)	10 (2.2)	20 (2.6)
Checkpoint inhibitor			
Nivolumab	16 (5.1)	32 (7.0)	48 (6.2)

*1L* first-line; *2L* second-line; *3L* third-line; *AE* adverse event; *IC0/1/2/3* PD-L1 expression level on tumor-infiltrating immune cells; *mTOR* mammalian target of rapamycin; *PD-L1* programmed death-ligand 1; *TKI* tyrosine kinase inhibitor; *VEGFR* vascular endothelial growth factor receptor

^a^Only therapies with prevalence ≥ 3% are presented

### OS by PD-L1 expression (stratified log-rank test)

**Fig. 1:**
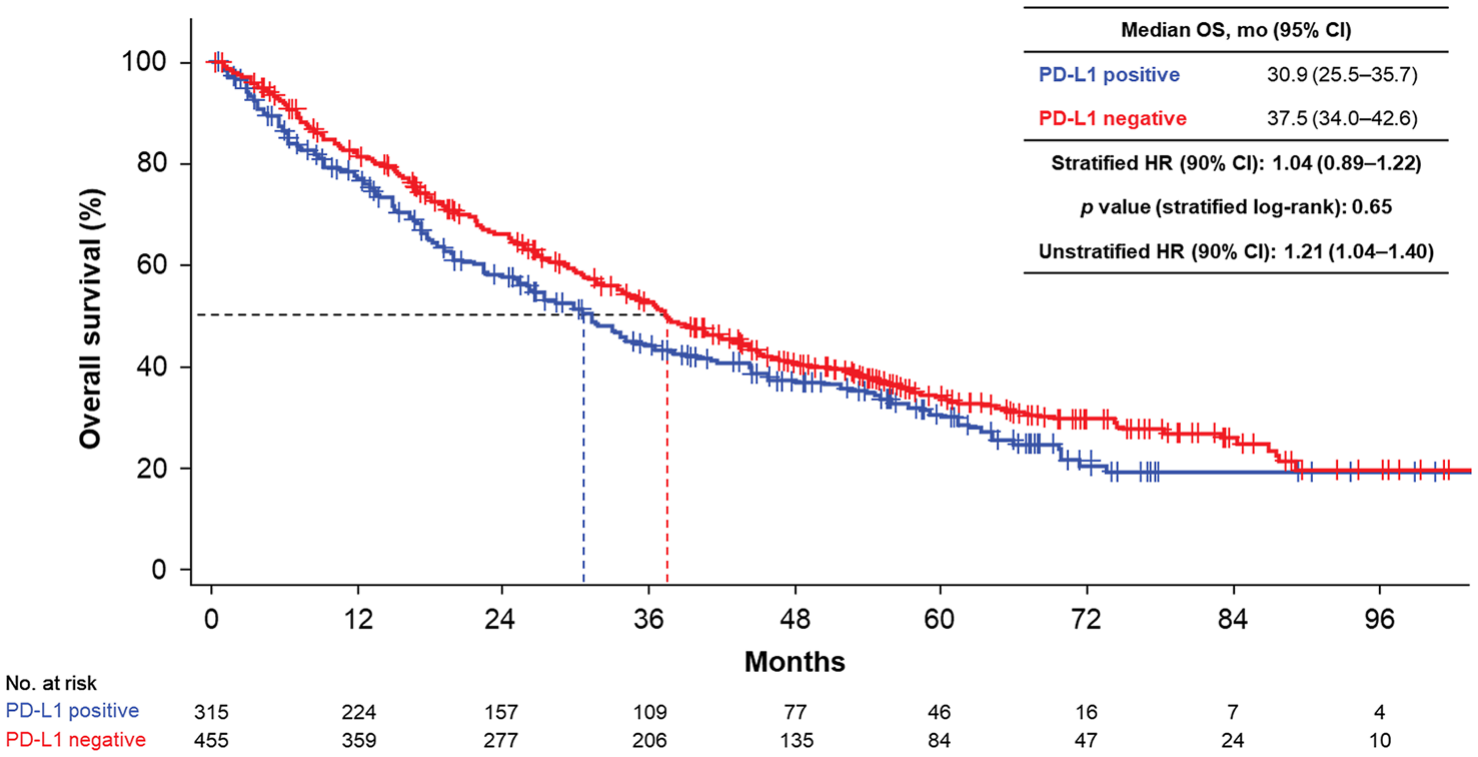
KM curves of OS by PD-L1 status. *CI* confidence interval; *HR* hazard ratio; *KM* Kaplan–Meier; *mo* months; *OS* overall survival; *PD-L1* programmed death-ligand 1

Median OS in PD-L1–positive and –negative patients was 30.9 (95% CI 25.5–35.7) and 37.5 (95% CI 34.0–42.6) months, respectively (stratified HR = 1.04 [90% CI 0.89–1.22, *p* = 0.65], unstratified HR 1.21 [90% CI 1.04–1.40]; Fig. 1). No statistically significant difference in OS distribution was observed; thus, the primary analysis suggested that PD-L1 status alone had no prognostic significance on outcomes in RCC.

### OS by PD-L1 expression (PSW–adjusted analysis)

**Fig. 2:**
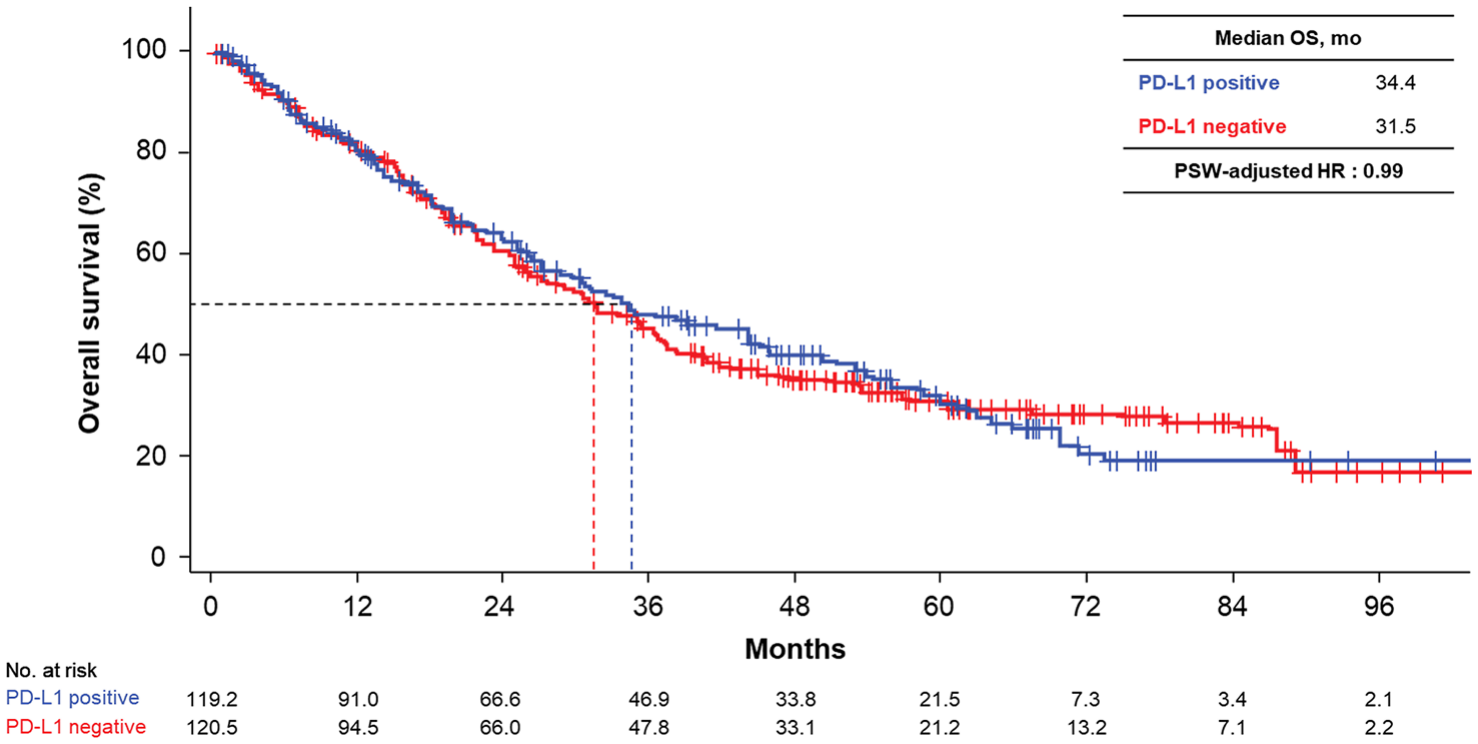
KM curves of PSW-adjusted OS. *HR* hazard ratio; *KM* Kaplan–Meier; *mo* months; *OS* overall survival; *PD-L1* programmed death-ligand 1; *PSW* propensity score-weighted

Sufficient overlap was observed in the distribution of estimated propensity scores. Histograms of pre- and post-PSW-adjusted distributions for PD-L1–positive and –negative groups are shown in Supplementary Fig. S1. No significant difference in PSW-adjusted OS was observed between the two groups, supporting the interpretation of the stratified analysis (Fig. 2). Median PSW-adjusted OS in the PD-L1–positive and –negative groups were 34.4 and 31.5 months, respectively (estimated PSW-adjusted HR 0.99). Additionally, HR stratified by propensity score per quartile was 1.045. Data pertaining to PSW analysis are shown in Supplementary Tables S4 and S5.

### OS by PD-L1 expression in subgroups

**Fig. 3:**
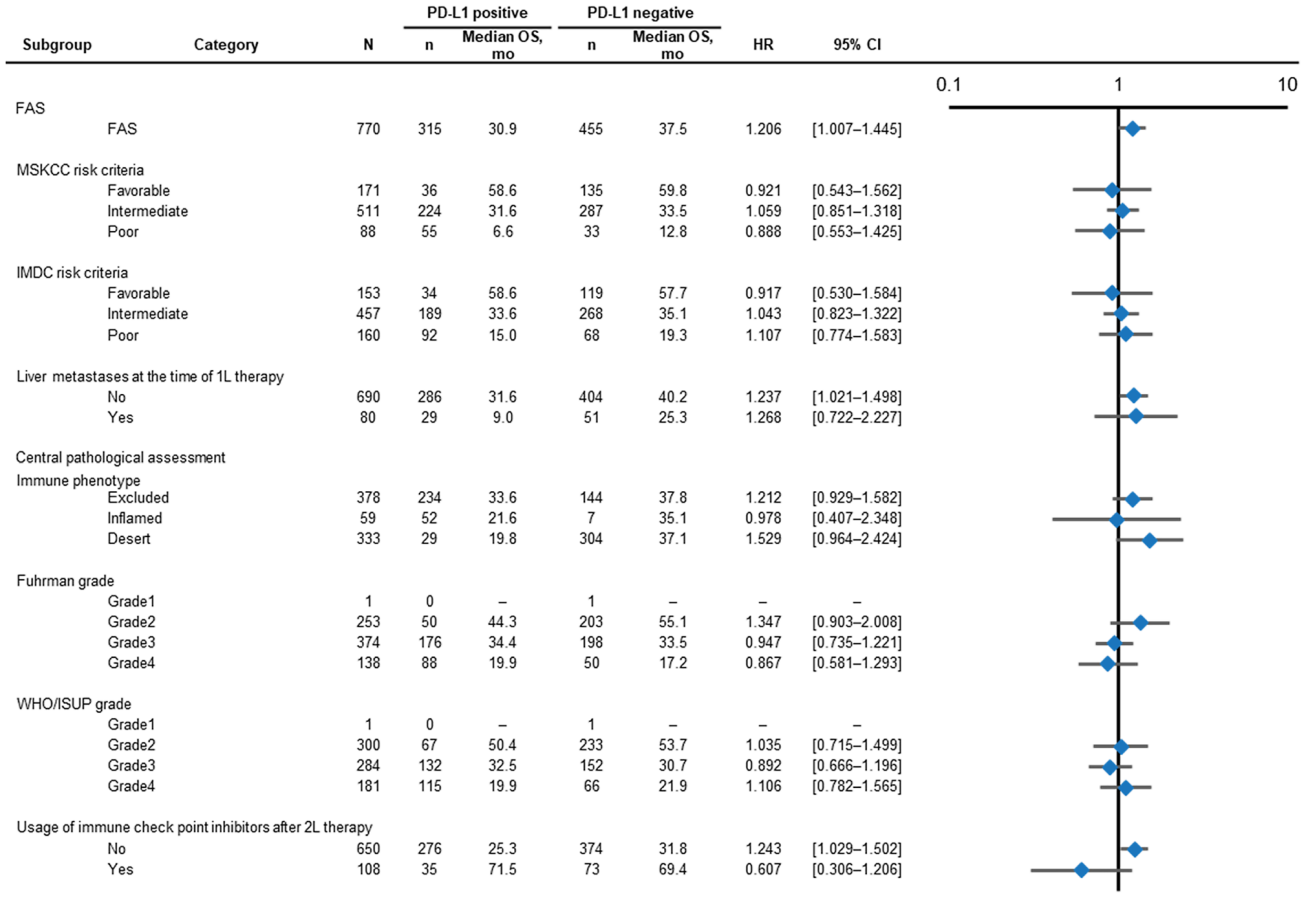
Forest plot of OS by PD-L1 status and patient subgroups. *CI* confidence interval; *FAS* full analysis set; *HR* hazard ratio; *IMDC* International Metastatic RCC Database Consortium; *mo* months; *MSKCC* Memorial Sloan Kettering Cancer Center; *OS* overall survival; *PD-L1* programmed death-ligand 1; *WHO/ISUP* World Health Organization/International Society of Urologic Pathologists

**Fig. 4:**
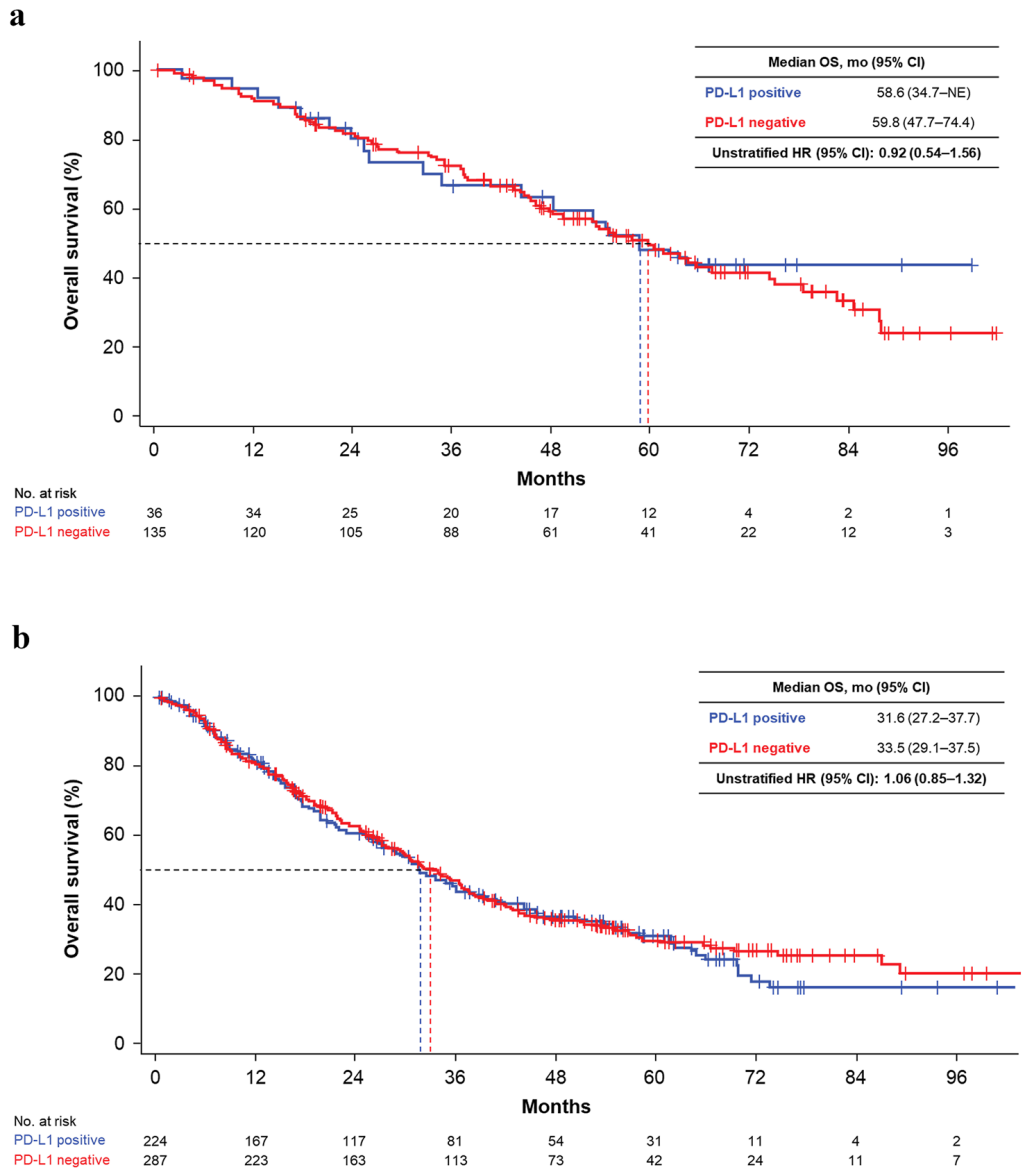

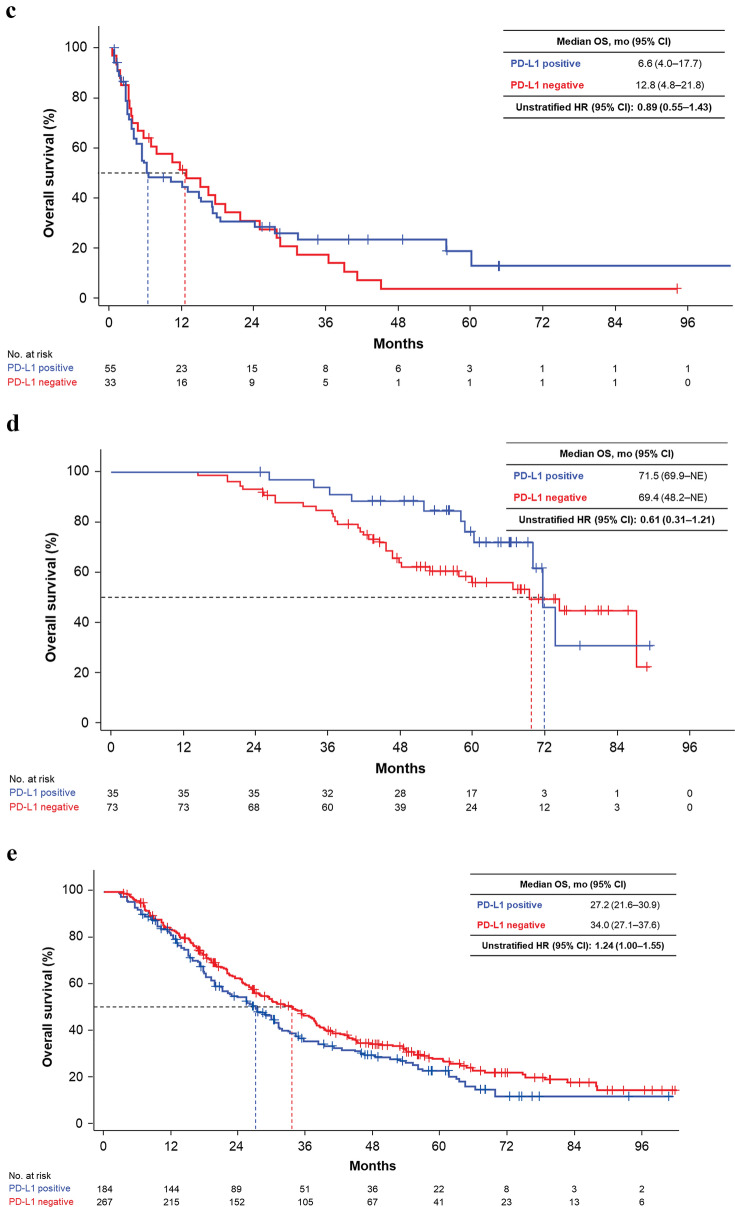
KM curves of OS by PD-L1 status in subgroups after treatment. Subgroups were MSKCC risk criteria **A** favorable, **B** intermediate, and **C** poor, and patients who **D** used and **E** did not use immune checkpoint inhibitor after 2L therapy. *2L* second-line; *CI* confidence interval; *HR* hazard ratio; *KM* Kaplan–Meier; *mo* months; *NE* not evaluable; *OS* overall survival; *PD-L1* programmed death-ligand 1

In subgroups based on MSKCC risk at 1L therapy, there was no significant difference in OS distribution based on PD-L1–positive or –negative status for favorable (median 58.6 versus 59.8 months; unstratified HR 0.92; 95% CI 0.54–1.56), intermediate (median 31.6 versus 33.5; HR 1.06; 95% CI 0.85–1.32) or poor (median 6.6 versus 12.8; unstratified HR 0.89; 95% CI 0.55–1.43) categories (Figs. 3 and 4). Subgroup analysis of OS by liver metastases status at the time of 1L therapy showed a trend towards shorter OS for PD-L1–positive versus PD-L1–negative patients (median OS for absent: 31.6 versus 40.2 months; unstratified HR 1.24; 95% CI 1.02–1.50, present: 9.0 versus 25.3; unstratified HR 1.27; 95% CI 0.72–2.23; Fig. 3, Supplementary Fig. S2). Results from other subgroups (based on nuclear grade, IMDC risk criteria, or immune phenotype; Fig. 3, Supplementary Fig. S3–S7) are also reported.

### OS by CPI treatment and PD-L1 status

Of 559 patients who received ≥ 2L therapy, 108 received nivolumab after 2L therapy (CPI-treated). Median OS observed for PD-L1–positive versus –negative patients in the CPI-treated group was 71.5 versus 69.4 months (unstratified HR 0.61; 95% CI 0.31–1.21) and 27.2 versus 34.0 months (unstratified HR 1.24; 95% CI 1.00–1.55) in the CPI-untreated group. In these two groups, KM curves showed a reverse trend (Fig. 4D, E). The analyses in patients who never received CPI throughout their treatment are shown in Supplementary Fig. S8.

### Treatment duration in 1L and 2L settings by PD-L1 status

Treatment duration in the 1L setting was slightly shorter in the PD-L1–positive group than in the PD-L1–negative group (median 5.1 months [95% CI 4.3–5.9] versus 6.0 months [95% CI 5.3–7.0]; unstratified HR 1.102 [95% CI 0.95–1.28]; stratified HR 1.01 [95% CI 0.87–1.17]; Supplementary Fig. S9a).

Treatment duration of 2L therapy was also shorter in the PD-L1–positive group compared with the PD-L1–negative group (median 4.2 months [95% CI 3.4–5.1] versus 5.7 months [95% CI 4.9–6.7]; unstratified HR 1.22 [95% CI 1.03–1.46]; stratified HR 1.17 [95% CI 0.97–1.40]; Supplementary Fig. S9b).

## Discussion

Baseline patient characteristics and treatment patterns were similar to those previously reported in a real-world setting in Japan. MSKCC risk status distribution was better than that in the previous report (favorable: 22.2% versus 13.2%; intermediate: 66.4% versus 68.1%; poor: 11.4% versus 18.7%) [23].

The proportion of PD-L1–positive patients (40.9%) is within the range reported previously (40–54%) [2, 24]. A higher prevalence of PD-L1 positivity was observed in patients with poor prognostic features (i.e., poor MSKCC/IMDC risk) and high-risk pathological features (higher clinical stage, higher nuclear grade, and sarcomatoid features). These observations are consistent with other reports in patients with high nuclear grade, sarcomatoid component, or high IMDC risk status; patients with clear cell RCC and poor risk status were more likely to express PD-L1 on TC than those with intermediate or favorable status (poor: 43%; intermediate: 29%; favorable: 17%; in METEOR study [NCT01865747]) [8, 9, 25]. PD-L1–positive patients showed more CD8-infiltrated phenotype than PD-L1–negative patients, which aligns with studies describing the correlation of IC status with T-effector gene signature [24].

Median OS after 1L treatment was 6 months shorter in the PD-L1–positive than in the –negative group, consistent with previous studies [9]. However, no statistically significant difference in OS distribution was observed between PD-L1–positive and –negative groups when stratified by MSKCC risk and liver metastases. The robustness of this primary analysis was supported by PSW–adjusted analysis. Additionally, in subgroups based on MSKCC risk, OS distributions were very similar for PD-L1–positive and –negative patients and were similarly shortened depending on risk status (Fig. 4a–c). This result aligns with those from the multivariable logistic regression model used to estimate the propensity score, which showed the highest odds ratio with MSKCC risk status, meaning it had the most relevant clinical covariates with PD-L1 expression (Supplementary Tables S2 and S3). MSKCC risk status distribution in PD-L1–positive patients was biased towards a poorer prognosis than in PD-L1–negative patients, suggesting that the OS difference between PD-L1–positive and –negative populations was mainly caused by difference of MSKCC risk status distribution. Therefore, PD-L1 status of primary lesion may not have clinical value when MSKCC/IMDC risk assessment is performed in recurrent/metastatic RCC in the era before CPIs became the standard of care for 1L treatment.

The role of PD-L1 expression as a biomarker for CPI response in RCC is not yet established due to its biological complexity (intra/inter patient-heterogeneity and dynamic nature of the marker). Recent studies and a meta-analysis found significantly improved OS and progression-free survival in CPI-treated PD-L1–positive patients [2, 3, 26]. In this study, OS was longer in PD-L1–positive patients who received ≥ 2L CPI therapy versus PD-L1–negative patients; the results were reversed in CPI-untreated patients.

New findings in the subgroup analysis by pathological features were observed. Previous studies have shown that low nuclear grades and low CD8 + T-cell infiltration are associated with better outcomes in RCC [13, 27]. This study found a trend towards shorter OS in PD-L1–positive patients with lower Fuhrman grades (e.g., Grade 2; median OS: 44.3 versus 55.1 months; unstratified HR 1.35 [95% CI 0.90–2.01]) and immune desert phenotype (median OS: 19.8 versus 37.1 months; unstratified HR 1.53 [95% CI 0.96–2.42]). Interestingly, this observation was not seen in the WHO/ISUP Grade 2 population (Supplementary Fig. S3a and S4a). Nonetheless, this finding suggests that prognosis worsens for patients with RCC if PD-L1–positive ICs infiltrate the tumor, even if other pathological characteristics were favorable. However, among patients with an inflamed phenotype and in subgroups with higher pathological grade, PD-L1–positive patients had similar OS compared with PD-L1-negative patients. These results suggest that a comprehensive examination of PD-L1 (IC) status, malignancy, and CD8 infiltration in surgical specimens may be needed when predicting prognosis.

Due to the retrospective nature of the study, unmeasured confounding factors could have had an effect. This study was based on mRCC after nephrectomy, which is representative of patients with RCC in the real world who have undergone nephrectomy but excludes those who have not. Because central pathologists only reviewed selected representative slides, some pathological features could have been underdiagnosed. Before 2009, the proportion of PD-L1–negative samples was greater than PD-L1–positive ones; however, the trend reversed between 2013 and 2015. A recent study found a strong correlation between high PD-L1 expression and tumor grade in non-small cell lung cancer [28]. In this study, PD-L1 positivity was higher in patients with high-risk pathological features (higher clinical stage, higher nuclear grade, and sarcomatoid features; Table 1), and the proportion of patients in Stage I/II was higher during the earlier collection period (Supplementary Table S6). Thus, the difference in PD-L1 positivity across sample collection years was likely to be a result of uneven clinical stage distribution rather than sample storage duration.

These results are clinically relevant in understanding the prognostic value of PD-L1 expression on recurrent/metastatic RCC before CPIs became the standard of care for 1L treatment. Overall, this retrospective study was the first to investigate potential associations between clinical outcomes and PD-L1 status in patients with previously treated RCC. Despite the retrospective nature of this study, our results suggest that PD-L1 status is not an independent prognostic factor in recurrent/metastatic RCC because, PD-L1 positivity was associated with other prognostic factors, especially MSKCC risk status.

## Supplementary Information

Below is the link to the electronic supplementary material.

Supplementary file1 (DOCX 1012 KB)
