# Correction to: Prognostic value of programmed death-ligand 1 status in Japanese patients with renal cell carcinoma

**DOI:** 10.1007/s10147-021-02008-5

**Published:** 2021-08-27

**Authors:** Motohide Uemura, Noboru Nakaigawa, Naoto Sassa, Katsunori Tatsugami, Kenichi Harada, Toshinari Yamasaki, Nobuaki Matsubara, Takuya Yoshimoto, Yuki Nakagawa, Tamaki Fukuyama, Mototsugu Oya, Nobuo Shinohara, Hirotsugu Uemura, Toyonori Tsuzuki

**Affiliations:** 1 Department of Urology, Osaka University Graduate School of Medicine, 2-2 Yamadaoka, Suita, Osaka 565-0871 Japan; 2 Department of Urology, Yokohama City University Graduate School of Medicine, 22-2 Seto, Kanazawa Ward, Yokohama, Kanagawa 236-0027 Japan; 3 Department of Urology, Nagoya University Graduate School of Medicine, Furocho, Chikusa Ward, Nagoya, Aichi 464-8601 Japan; 4 Department of Urology, Kyushu University Graduate School of Medical Sciences, 3-1-1, Maidashi, Higashi-ku, Fukuoka City, 812-8582 Japan; 5 Division of Urology, Department of Surgery Related, Kobe University Graduate School of Medicine, 7-5-2, Kusunoki-cho, Chuo-ku, Kobe, Hyogo 650-0017 Japan; 6 Department of Urology, Kyoto University Graduate School of Medicine, Yoshidakonoecho, Sakyo Ward, Kyoto, 606-8501 Japan; 7 Department of Breast and Medical Oncology, National Cancer Center Hospital East, 6-5-1 Kashiwanoha, Kashiwa, Chiba 277-8577 Japan; 8 Biometrics Department, Chugai Pharmaceutical Co., Ltd., Nihonbashi Muromachi 2-1-1, Chuo City, Tokyo 103-8324 Japan; 9 Medical Affairs Division, Chugai Pharmaceutical Co., Ltd., Nihonbashi Muromachi 2-1-1, Chuo City, Tokyo 103-8324 Japan; 10 Department of Urology, Keio University School of Medicine, 35 Shinanomachi, Shinjuku City, Tokyo 160-8582 Japan; 11 Department of Renal and Genitourinary Surgery, Hokkaido University Graduate School of Medicine, Kita 15, Nishi 7, Kita-ku, Sapporo, 060-8638 Japan; 12 Department of Urology, Kindai University, Faculty of Medicine, 377‐2 Ohnohigashi, Osaka-Sayama City, Osaka 589‐8511 Japan; 13 Department of Surgical Pathology, Aichi Medical University Hospital, 1-1 Yazakokarimata, Nagakute, Aichi 480-1195 Japan; 14 Present Address: Department of Urology, Aichi Medical University Hospital, 1-1 Yazakokarimata, Nagakute, Aichi 480-1195 Japan; 15 Present Address: Department of Urology, Kitakyushu Municipal Medical Center, 2-1-1 Bashaku, Kokurakita Ward, Kitakyushu, Fukuoka 802-0077 Japan

## Correction to: International Journal of Clinical Oncology https://doi.org/10.1007/s10147-021-01993-x

The article Prognostic value of programmed death‑ligand 1 status in Japanese patients with renal cell carcinoma, written by Motohide Uemura, Noboru Nakaigawa, Naoto Sassa, Katsunori Tatsugami, Kenichi Harada, Toshinari Yamasaki, Nobuaki Matsubara, Takuya Yoshimoto, Yuki Nakagawa, Tamaki Fukuyama, Mototsugu Oya, Nobuo Shinohara, Hirotsugu Uemura, and Toyonori Tsuzuki was originally published Online First without Open Access.

With the author(s)’ decision to opt for Open Choice the copyright of the article changed on August 18, 2021 to © Author(s) 2021 and the article is forthwith distributed under a Creative Commons Attribution 4.0 International License, which permits use, sharing, adaptation, distribution and reproduction in any medium or format, as long as you give appropriate credit to the original author(s) and the source, provide a link to the Creative Commons licence, and indicate if changes were made. The images or other third party material in this article are included in the article’s Creative Commons licence, unless indicated otherwise in a credit line to the material. If material is not included in the article’s Creative Commons licence and your intended use is not permitted by statutory regulation or exceeds the permitted use, you will need to obtain permission directly from the copyright holder. To view a copy of this licence, visit http://creativecommons.org/licenses/by/4.0.

The original article has been corrected.

