# Supplementary material for: Prognostic value of programmed death-ligand 1 status in Japanese patients with renal cell carcinoma
Source: Int J Clin Oncol. 2021 Jul 21;26(11):2073–84. doi: 10.1007/s10147-021-01993-x (PMC8520884; doi:10.1007/s10147-021-01993-x)
Supplement: Supplementary file 1 — Supplementary file1 (DOCX 1012 KB) [file 10147_2021_1993_MOESM1_ESM.docx]

**Prognostic Value of Programmed Death-Ligand 1 Status in Japanese Patients With Renal Cell Carcinoma**

**Running Title: Prognostic value of PD-L1 in RCC**

Motohide Uemura, MD PhD^1^; Noboru Nakaigawa, MD PhD^2^; Naoto Sassa, MD PhD^3,a^; Katsunori Tatsugami, MD PhD^4, b^; Kenichi Harada, MD PhD^5^; Toshinari Yamasaki, MD PhD^6^; Nobuaki Matsubara, MD^7^; Takuya Yoshimoto, PhD^8^; Yuki Nakagawa, MS^8^; Tamaki Fukuyama, MS^9^; Mototsugu Oya, MD PhD^10^; Nobuo Shinohara, MD PhD^11^; Hirotsugu Uemura, MD PhD^12^; Toyonori Tsuzuki, MD PhD^13^

**Affiliations:**

^1^Osaka University Graduate School of Medicine, Department of Urology, 2-2 Yamadaoka, Suita, Osaka 565-0871, Japan

^2^Yokohama City University Graduate School of Medicine, Department of Urology, 22-2 Seto, Kanazawa Ward, Yokohama, Kanagawa 236-0027, Japan

^3^Nagoya University Graduate School of Medicine, Department of Urology, Furocho, Chikusa Ward, Nagoya, Aichi 464-8601, Japan

^4^Kyushu University Graduate School of Medical Sciences, Department of Urology, 3-1-1, Maidashi, Higashi-ku, Fukuoka City, 812-8582 Japan

^5^Kobe University Graduate School of Medicine, Division of Urology, Department of Surgery Related, 7-5-2, Kusunoki-cho, Chuo-ku, Kobe, Hyogo 650-0017, Japan

^6^Kyoto University Graduate School of Medicine, Department of Urology, Yoshidakonoecho, Sakyo Ward, Kyoto, 606-8303, Japan

^7^National Cancer Center Hospital East, Department of Breast and Medical Oncology, 6-5-1 Kashiwanoha, Kashiwa, Chiba 277-8577, Japan

^8^Chugai Pharmaceutical Co., Ltd., Biometrics Department, Nihonbashi Muromachi 2-1-1, Chuo City, Tokyo 103-8324, Japan

^9^Chugai Pharmaceutical Co., Ltd., Medical affairs Division, Nihonbashi Muromachi 2-1-1, Chuo City, Tokyo 103-8324, Japan

^10^Keio University School of Medicine, Department of Urology, 35 Shinanomachi, Shinjuku City, Tokyo 160-8582, Japan

^11^Hokkaido University Graduate School of Medicine, Department of Renal and Genitourinary surgery, Kita 15, Nishi 7, Kita-ku, Sapporo,060-8638, Japan

^12^Kindai University Faculty of Medicine, Department of Urology, 377‐2 Ohnohigashi, Osaka-Sayama City, Osaka 589‐8511, Japan

^13^Aichi Medical University Hospital, Department of Surgical Pathology, 1-1 Yazakokarimata, Nagakute, Aichi 480-1195, Japan

^a^Current affiliation is Aichi Medical University Hospital, Department of Urology, 1-1 Yazakokarimata, Nagakute, Aichi 480-1195, Japan

^b^Current affiliation is Kitakyushu Municipal Medical Center, Department of Urology, 2-1-1 Bashaku, Kokurakita Ward, Kitakyushu, Fukuoka 802-0077, Japan

**Corresponding Author:** Hirotsugu Uemura, Kindai University Faculty of Medicine, Department of Urology, 377-2 Ohnohigashi, Osaka-sayama, Osaka 589-8511, Japan. Phone: +81-72-366-0221; Fax: +81-72-365-6273; E-mail: uemura@med.kindai.ac.jp

**Supplementary Figures**

**Fig. S1** Histograms of pre-/post-weighted propensity score distributions in PD-L1–negative and positive groups. PD-L1, programmed death-ligand 1

**
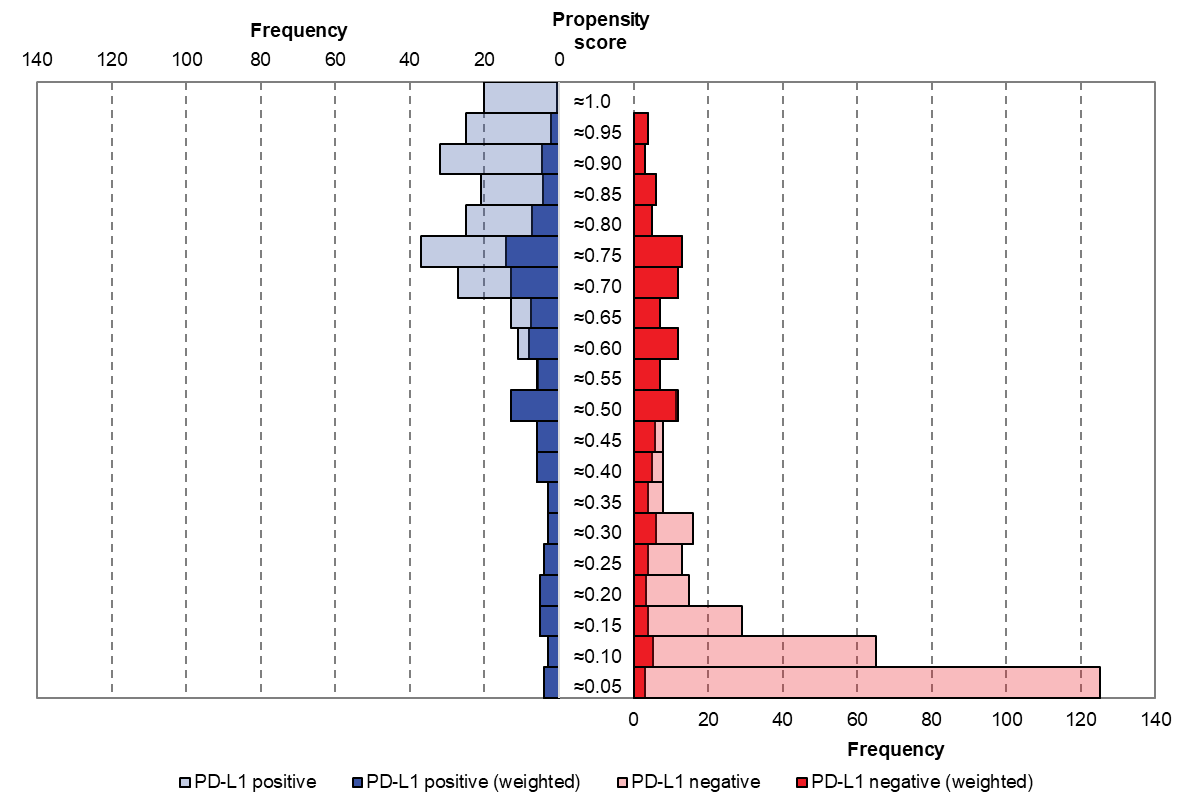
**

**Fig. S2** KM curves of OS in liver metastases (a) present and (b) absent subgroups by PD-L1 status. CI, confidence interval; HR, hazard ratio; KM, Kaplan-Meier; OS, overall survival; PD-L1, programmed death-ligand 1

**a**

**
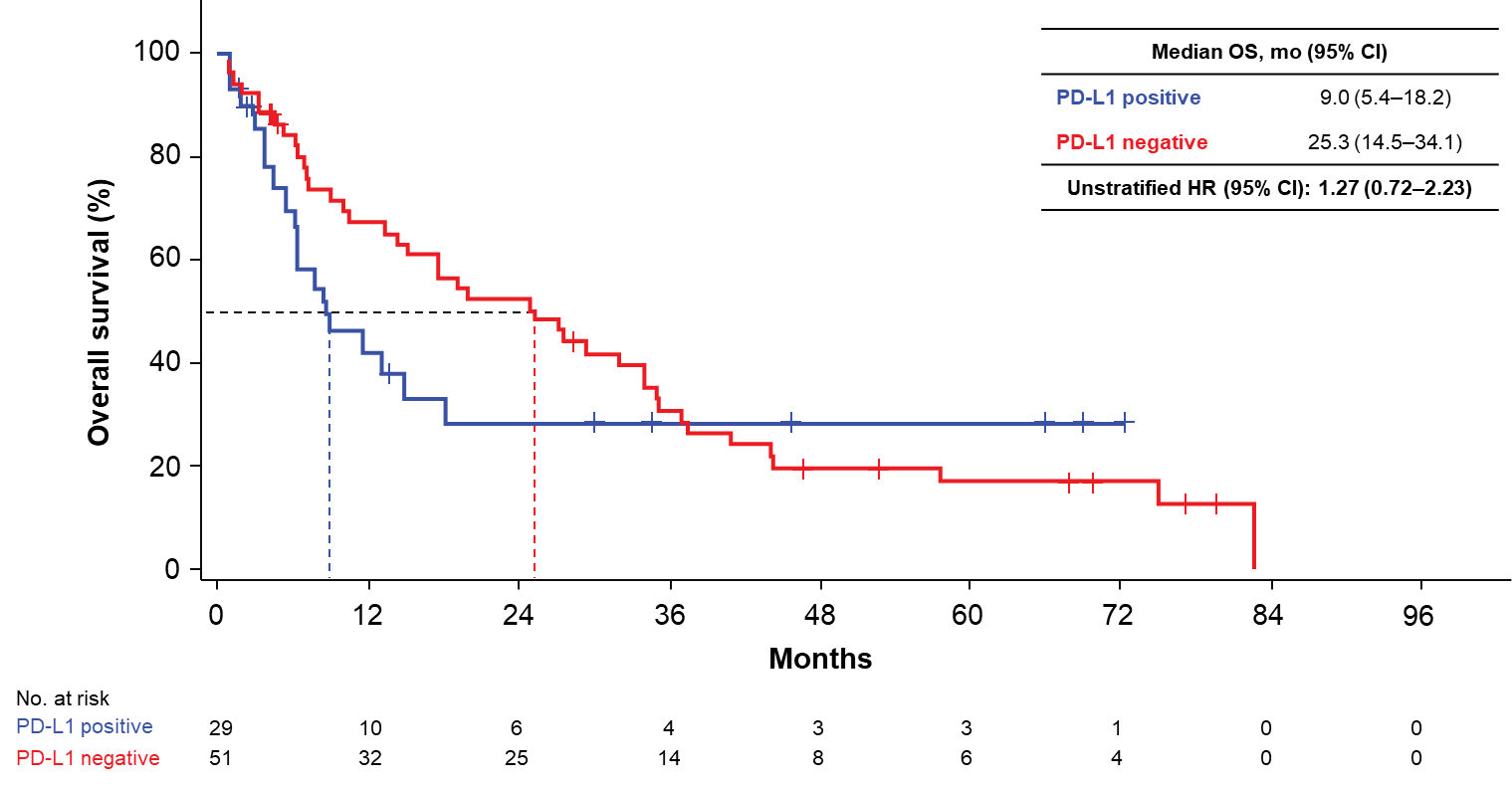
**

**b**

**
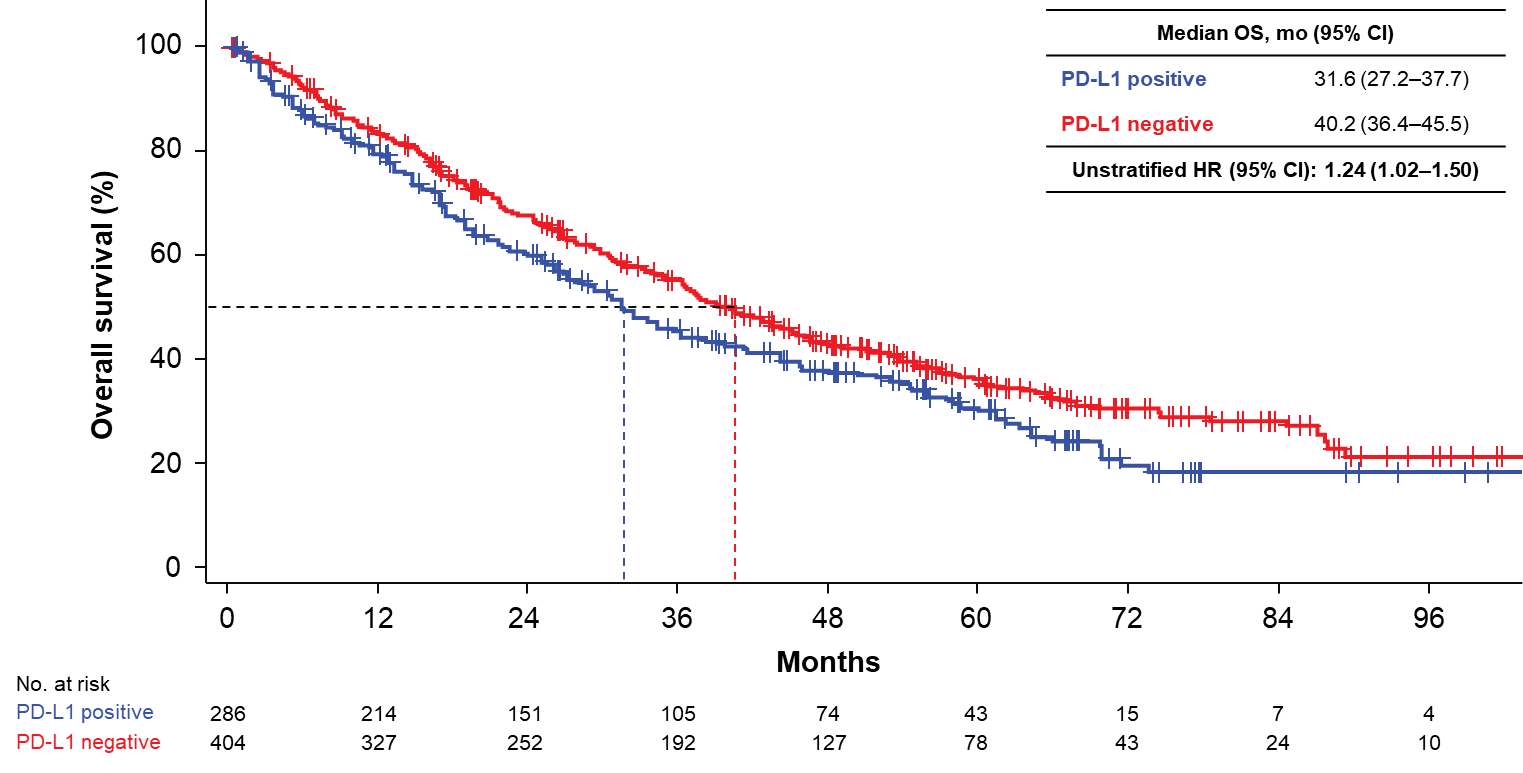
**

**Fig. S3** KM curves of OS in Fuhrman (a) grade 2, (b) grade 3, and (c) grade 4 subgroups by PD-L1 status. CI, confidence interval; HR, hazard ratio; KM, Kaplan-Meier; OS, overall survival; PD-L1, programmed death-ligand 1

**a**

**
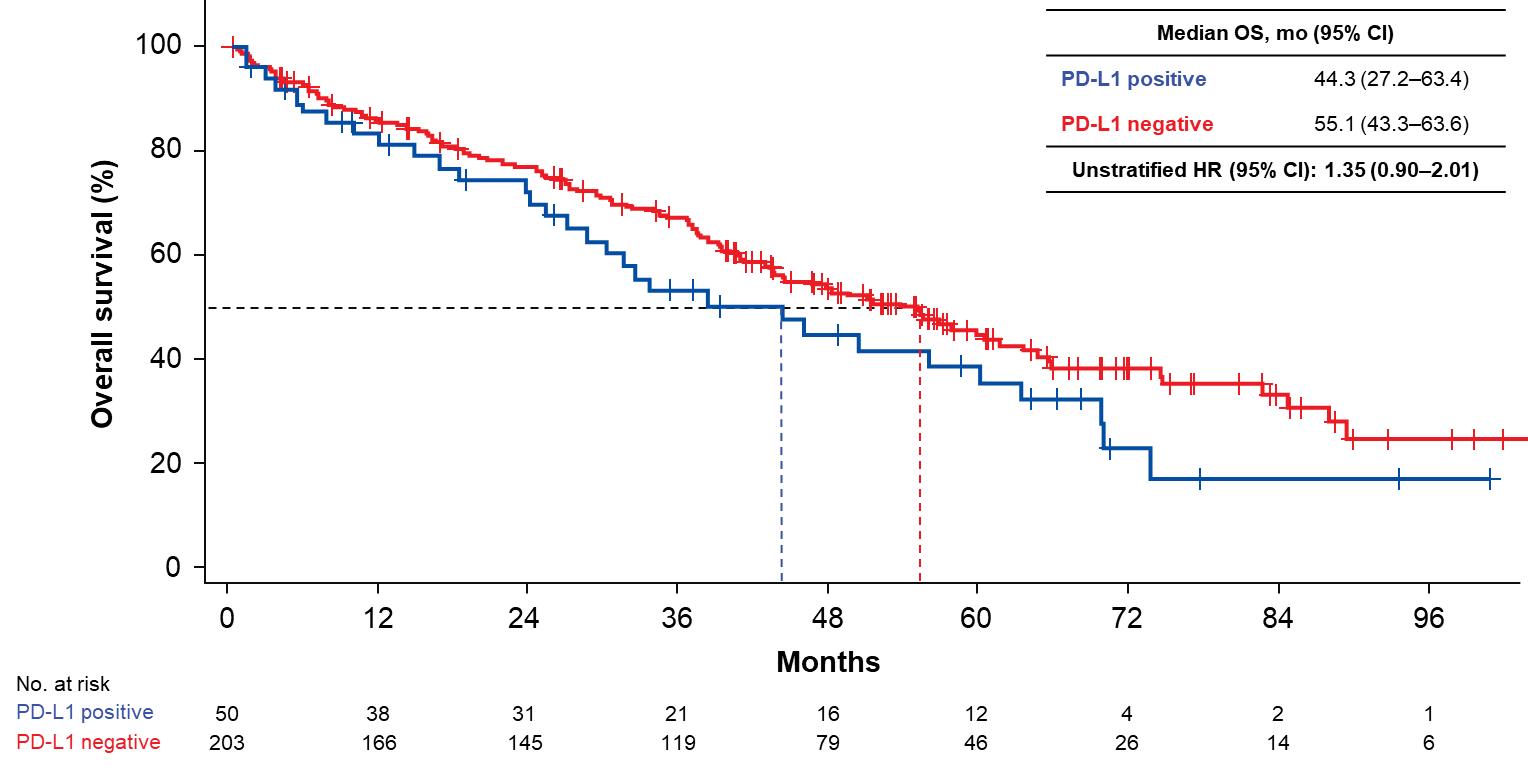
**

**b**

**
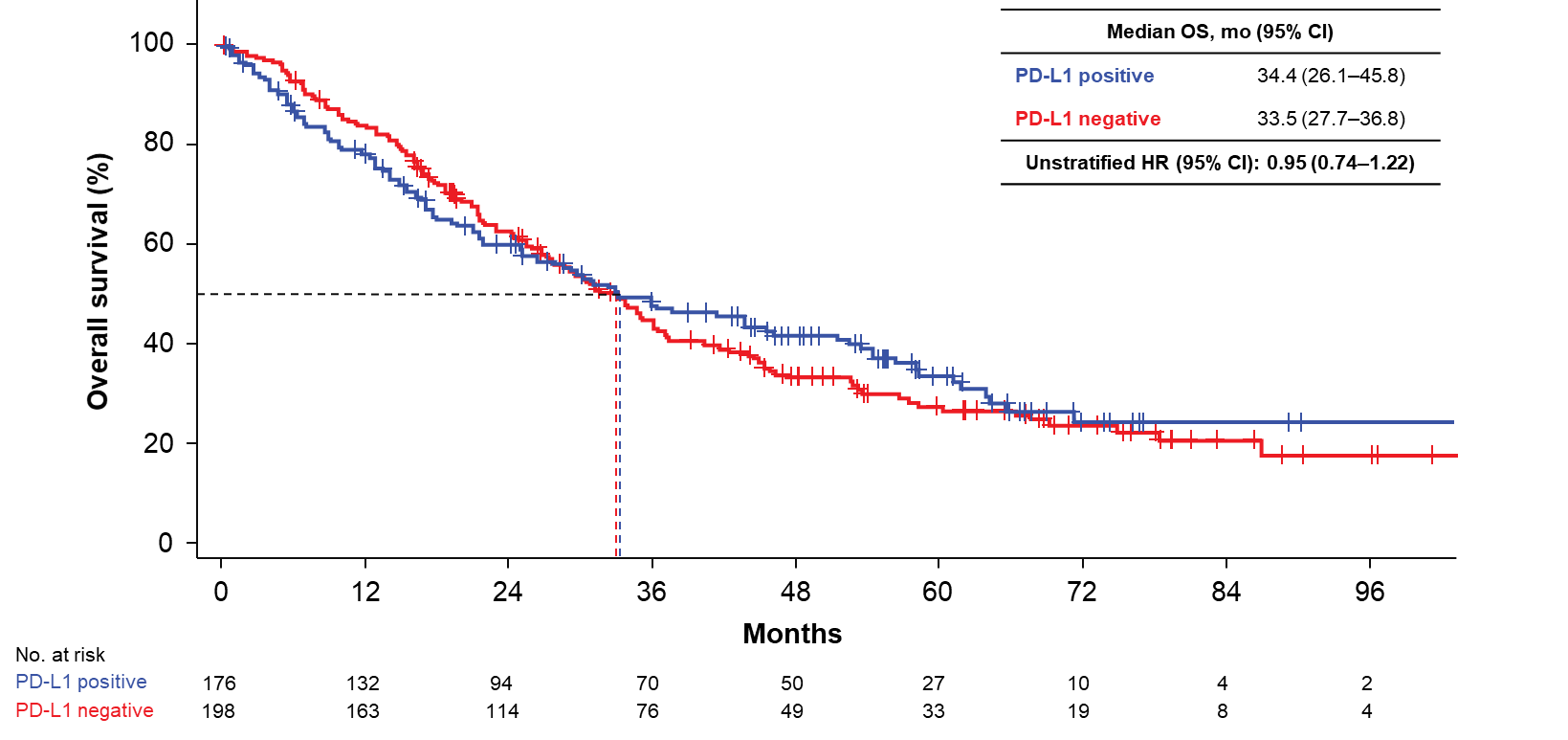
**

**c**

**
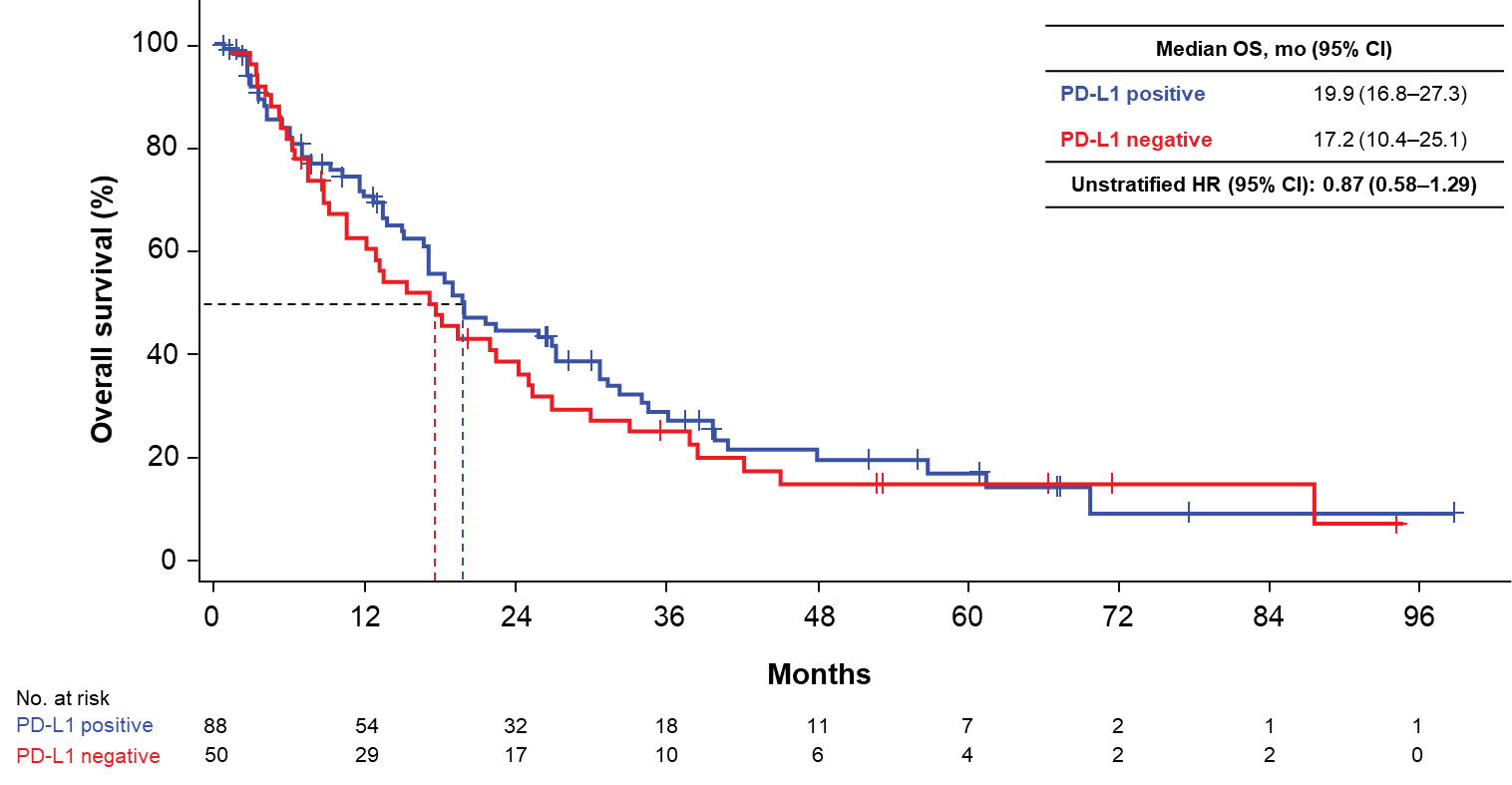
**

**Fig. S4** KM curves of OS in WHO/ISUP (A) grade 2, (B) grade 3, and (C) grade 4 subgroups by PD-L1 status. CI, confidence interval; HR, hazard ratio; KM, Kaplan-Meier; OS, overall survival; PD-L1, programmed death-ligand 1; WHO/ISUP, World Health Organization/International Society of Urologic Pathologists

**a**

**
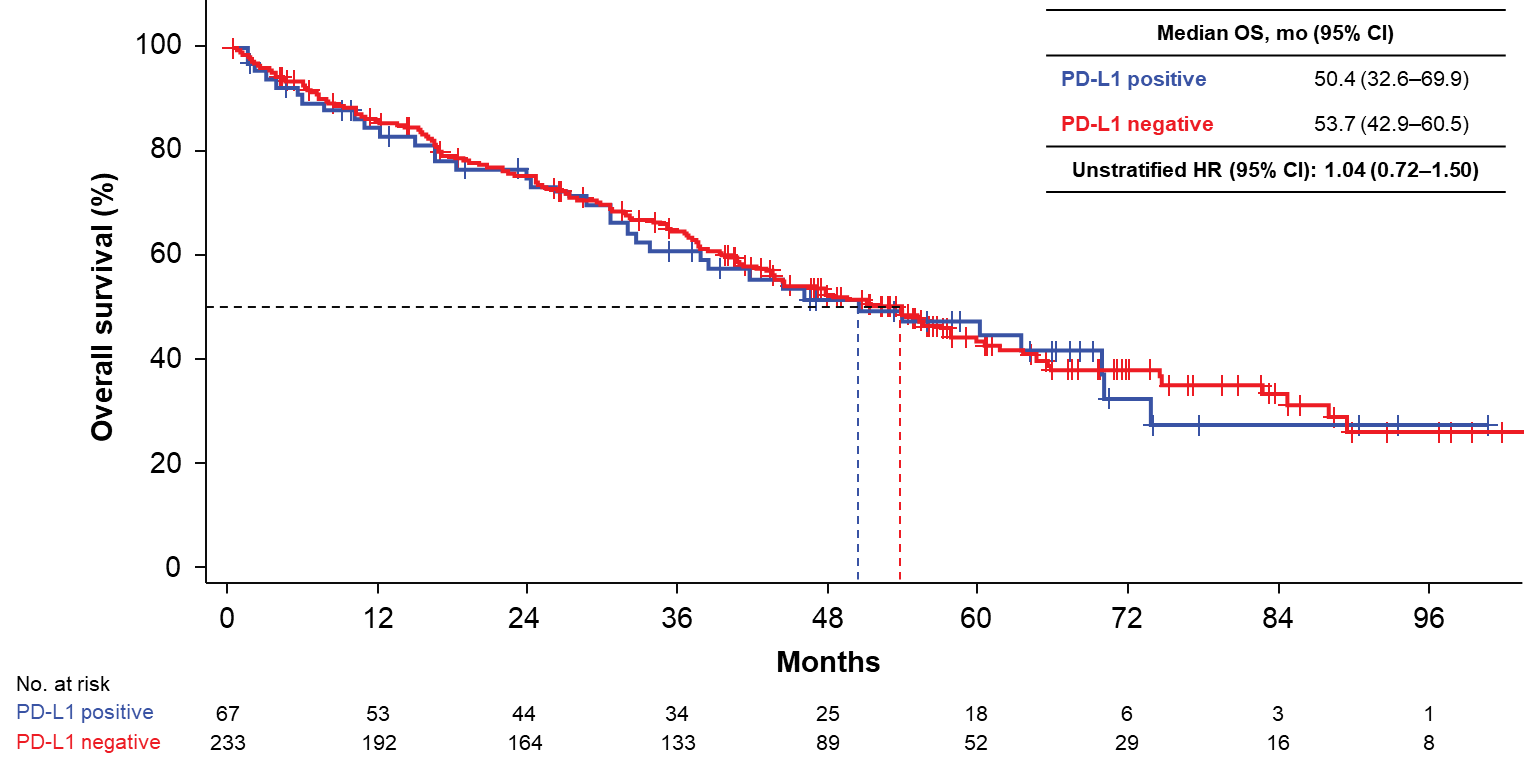
**

**b**

**
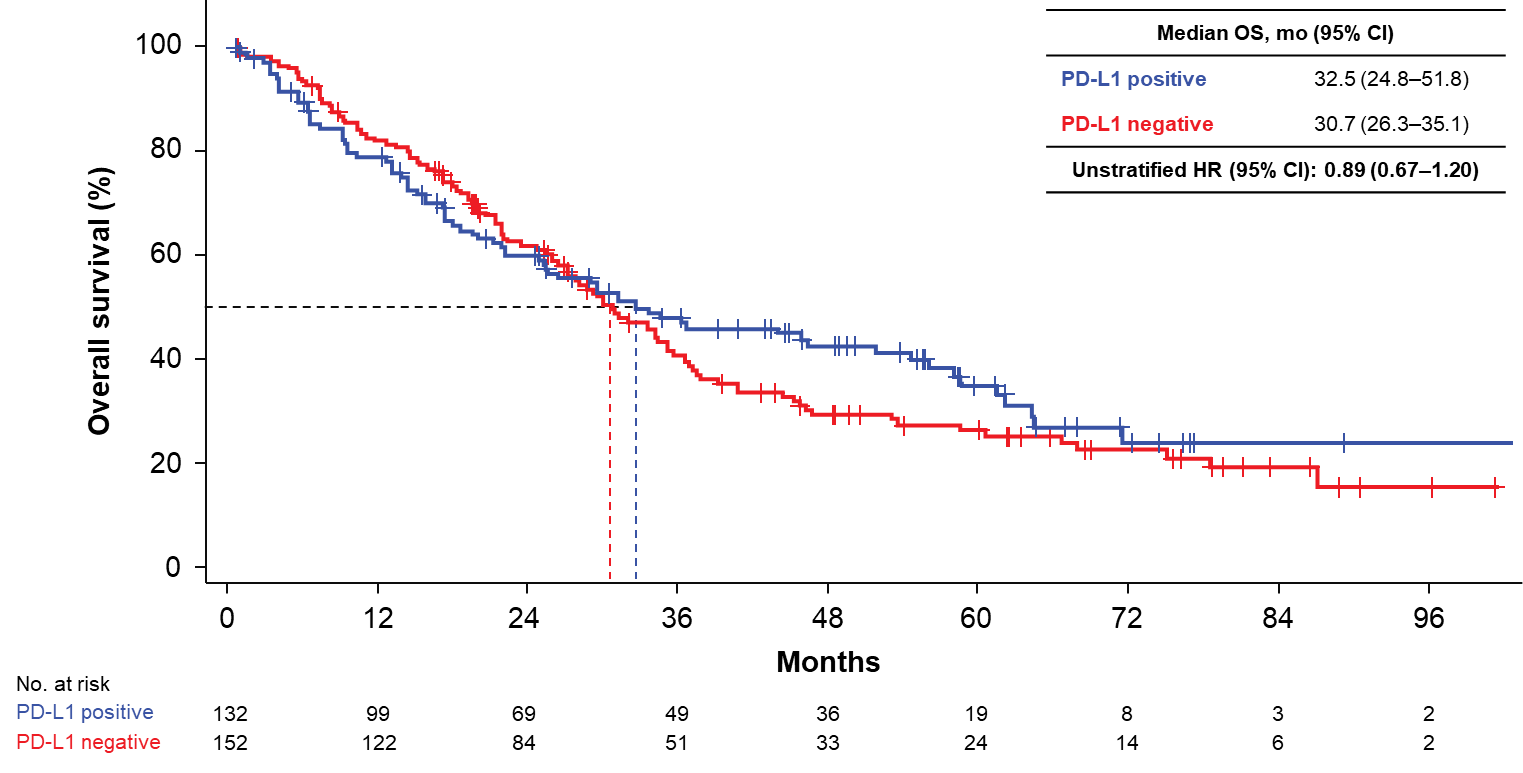
**

**c**

**
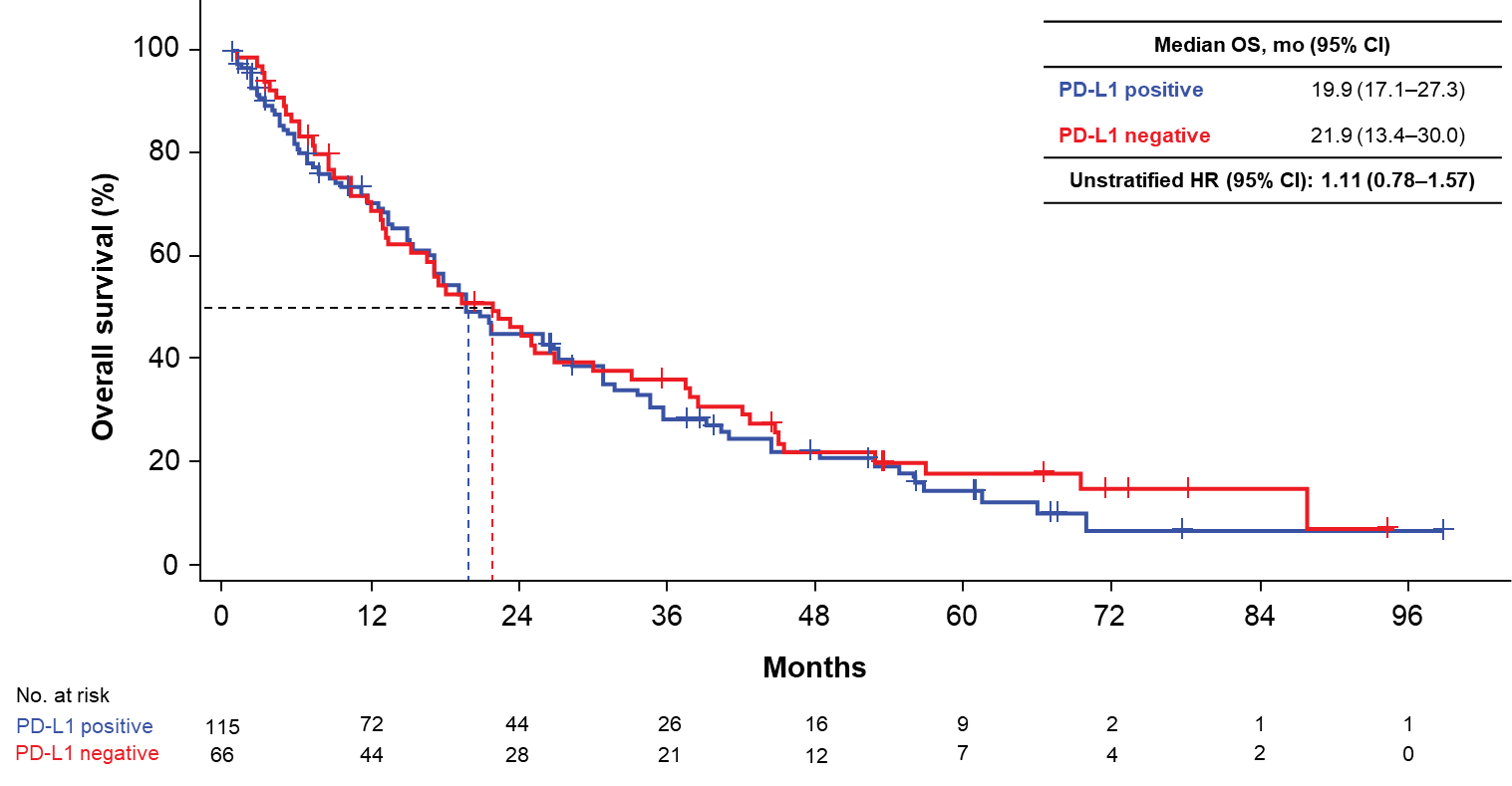
**

**Fig. S5** KM curves of OS in IMDC risk criteria (a) favorable, (b) intermediate, and (c) poor subgroups by PD-L1 status. CI, confidence interval; HR, hazard ratio; IMDC, International Metastatic Renal Cell Carcinoma Database Consortium; KM, Kaplan-Meier; NE, not evaluable OS, overall survival; PD-L1, programmed death-ligand 1

**a**

**
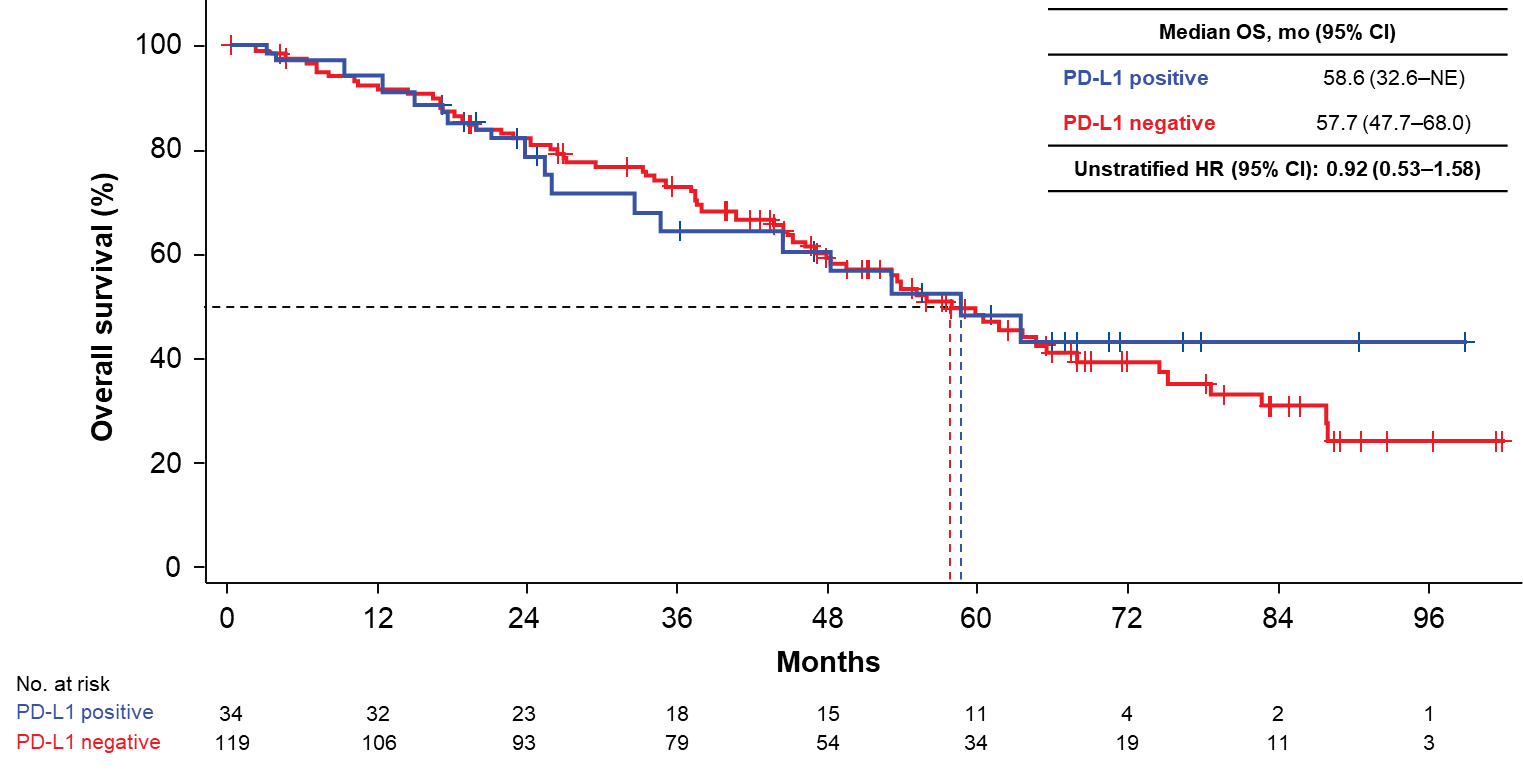
**

**b**

**
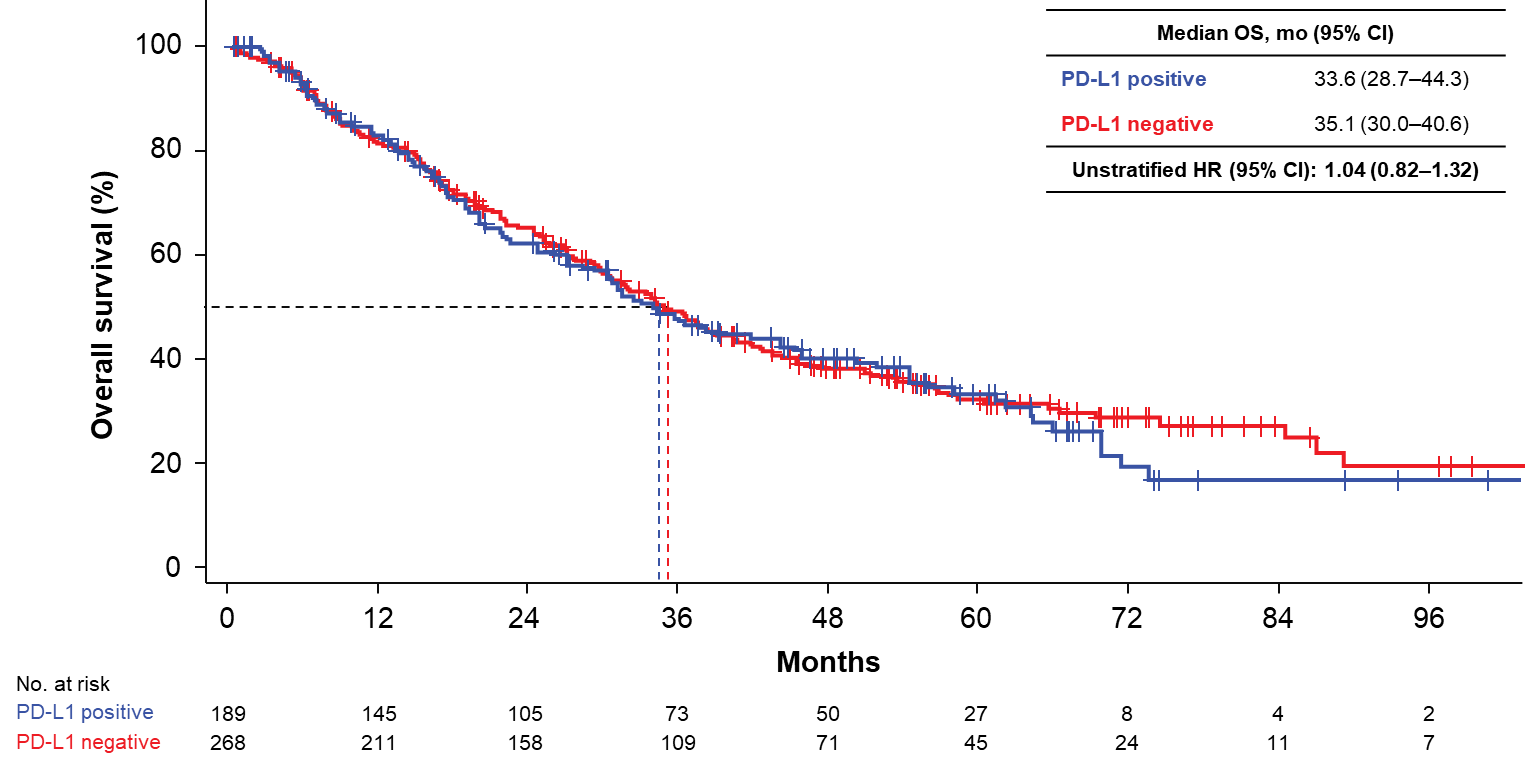
**

**c**

**
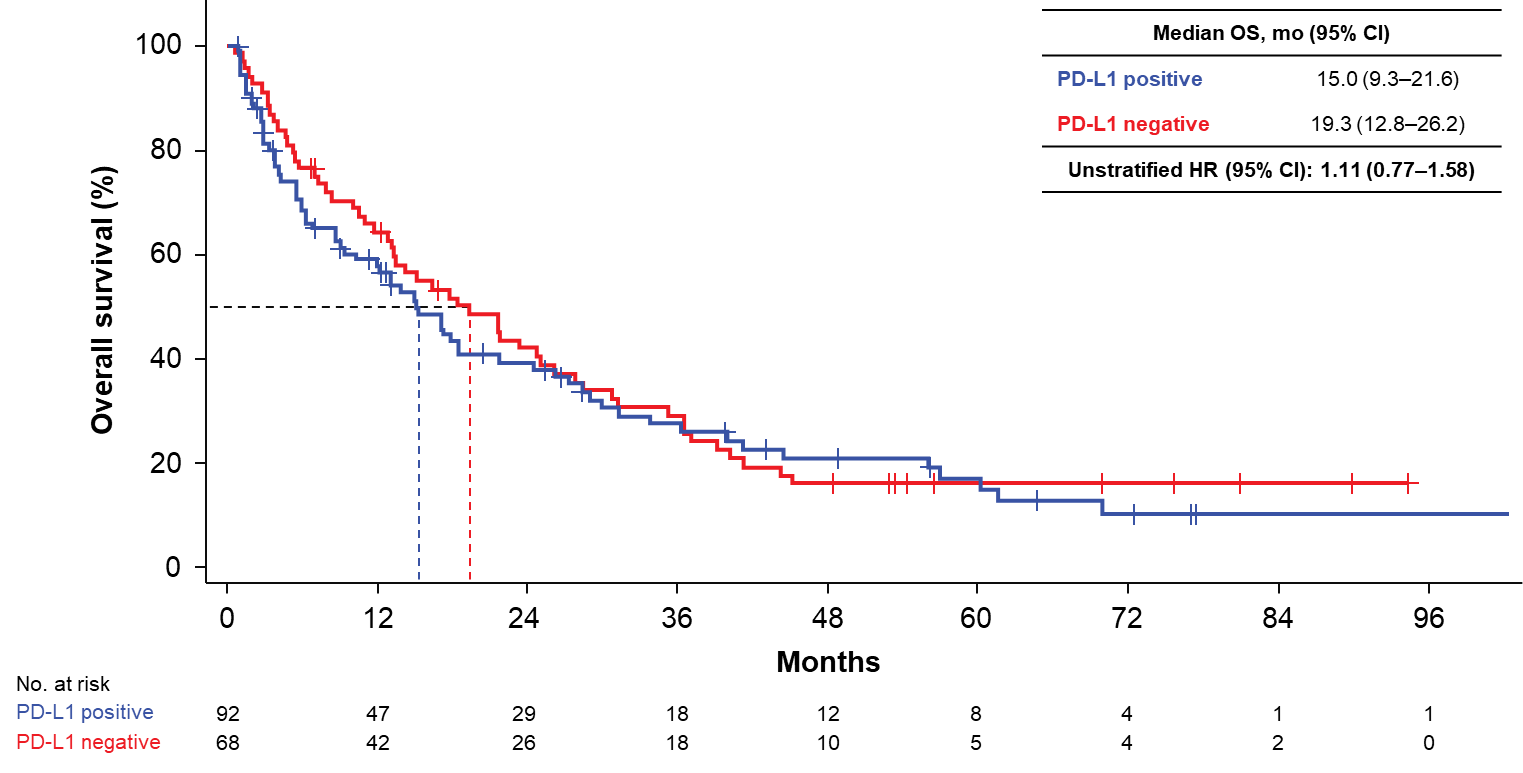
**

**Fig. S6** KM curves of OS in immune phenotype (a) excluded, (b) inflamed, and (c) desert subgroups by PD-L1 status. CI, confidence interval; HR, hazard ratio; KM, Kaplan-Meier; OS, overall survival; PD-L1, programmed death-ligand 1

**a**

**
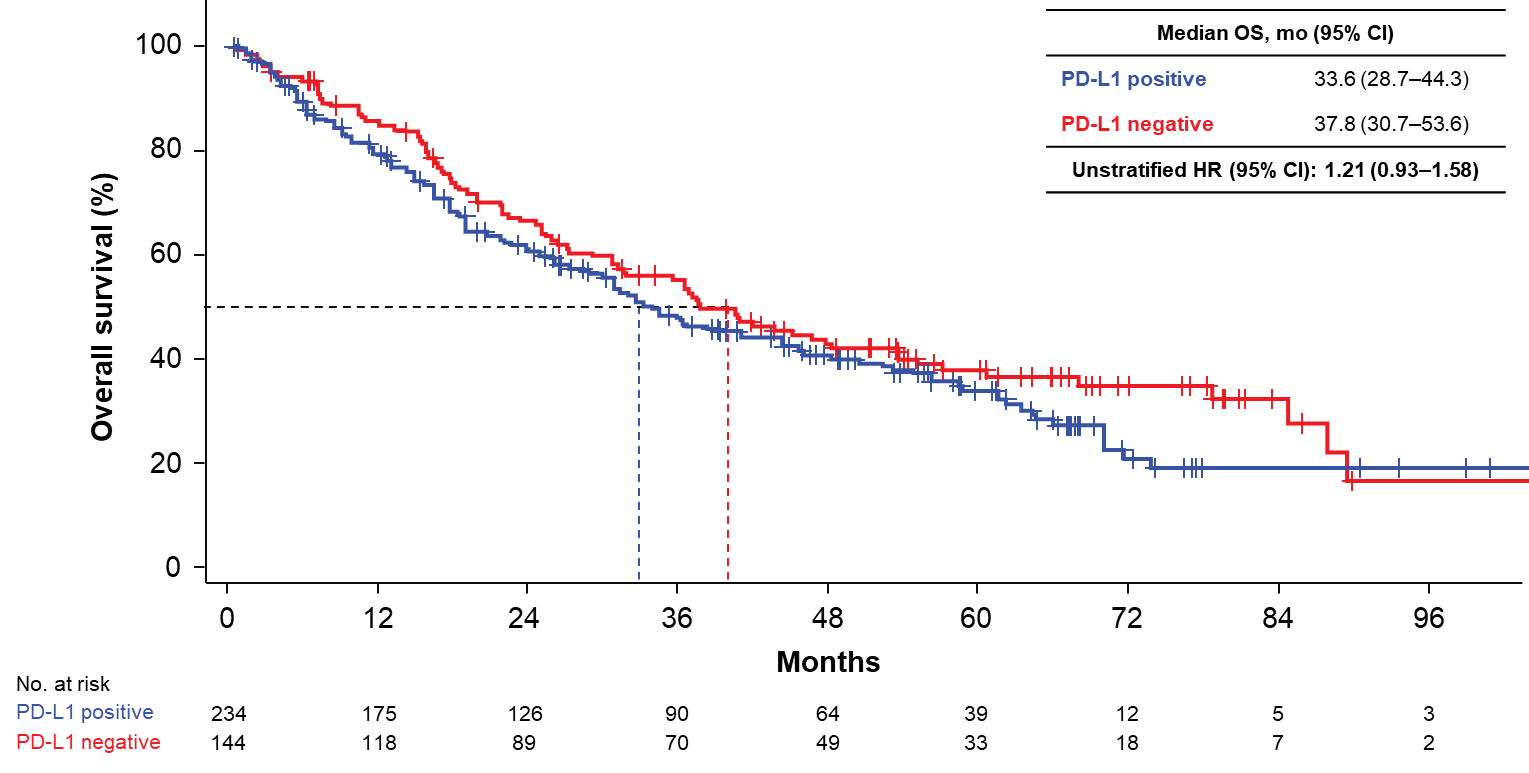
**

**b**

**
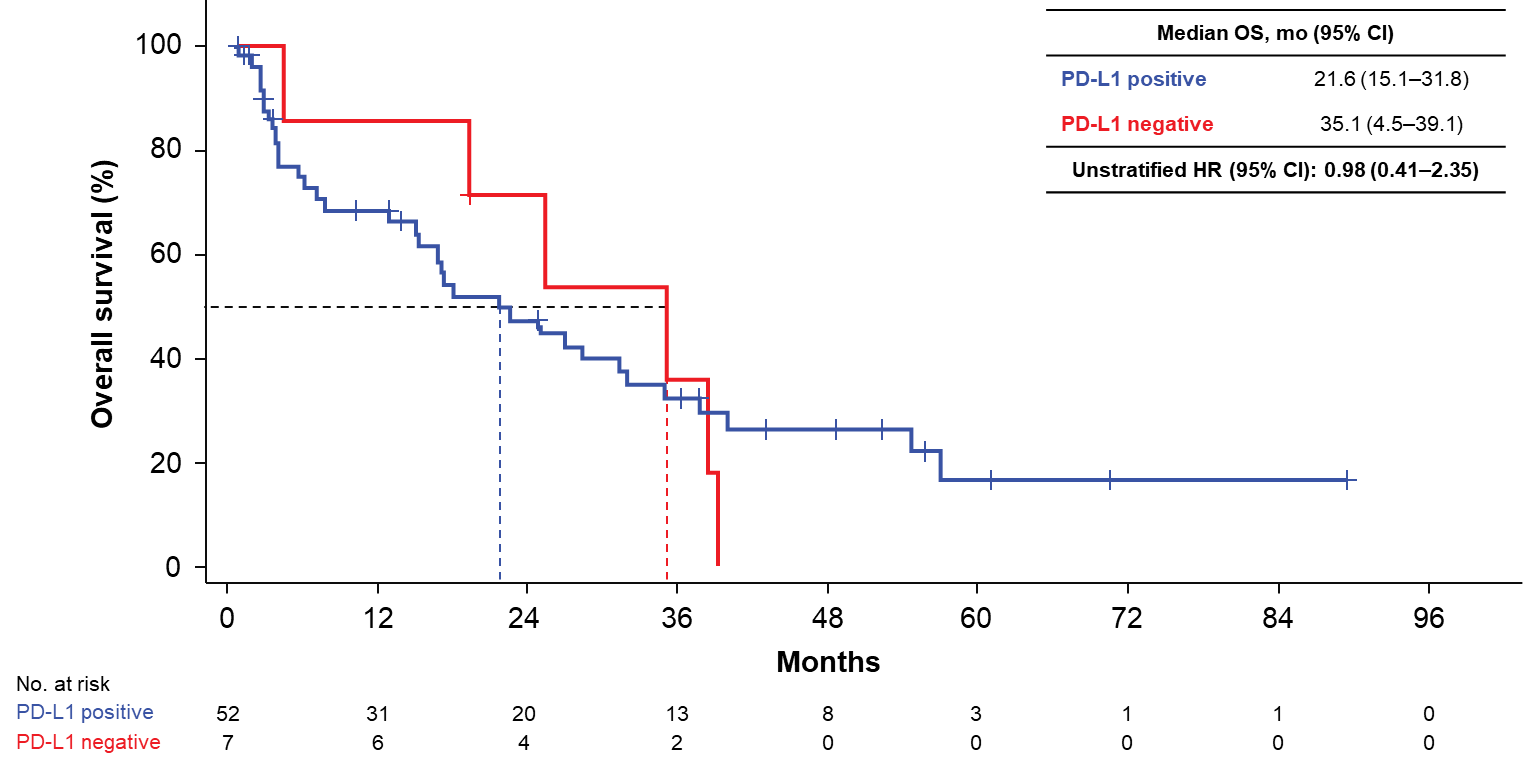
**

**c**

**
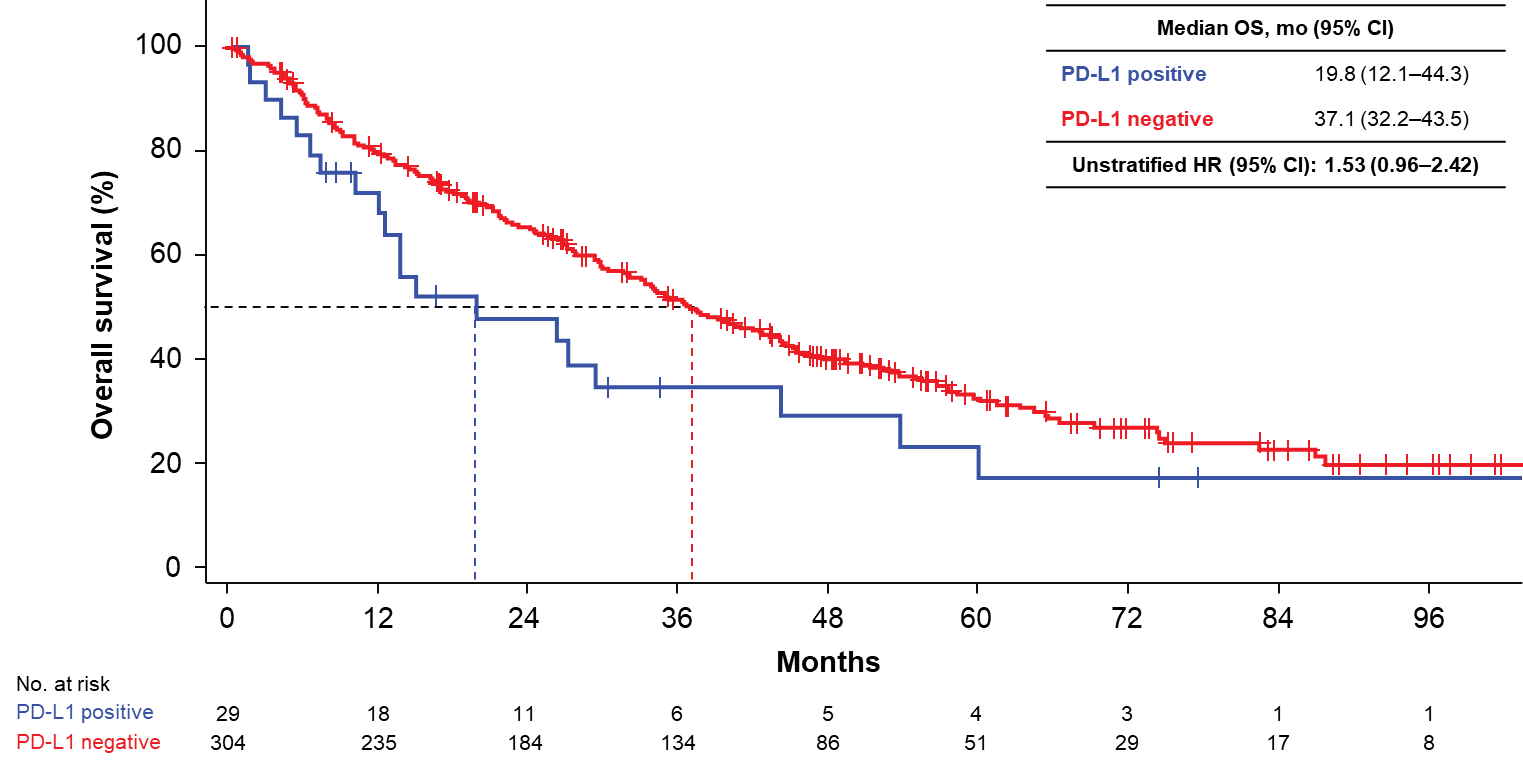
**

**Fig. S7** KM curves of OS by PD-L1 expression (IC0 vs IC1 vs IC2 vs IC3). ^a^ With respect to IC0. CI, confidence interval; HR, hazard ratio; IC0/1/2/3, PD-L1 expression level on tumor-infiltrating immune cells; KM, Kaplan-Meier; OS, overall survival; PD-L1, programmed death-ligand 1

**
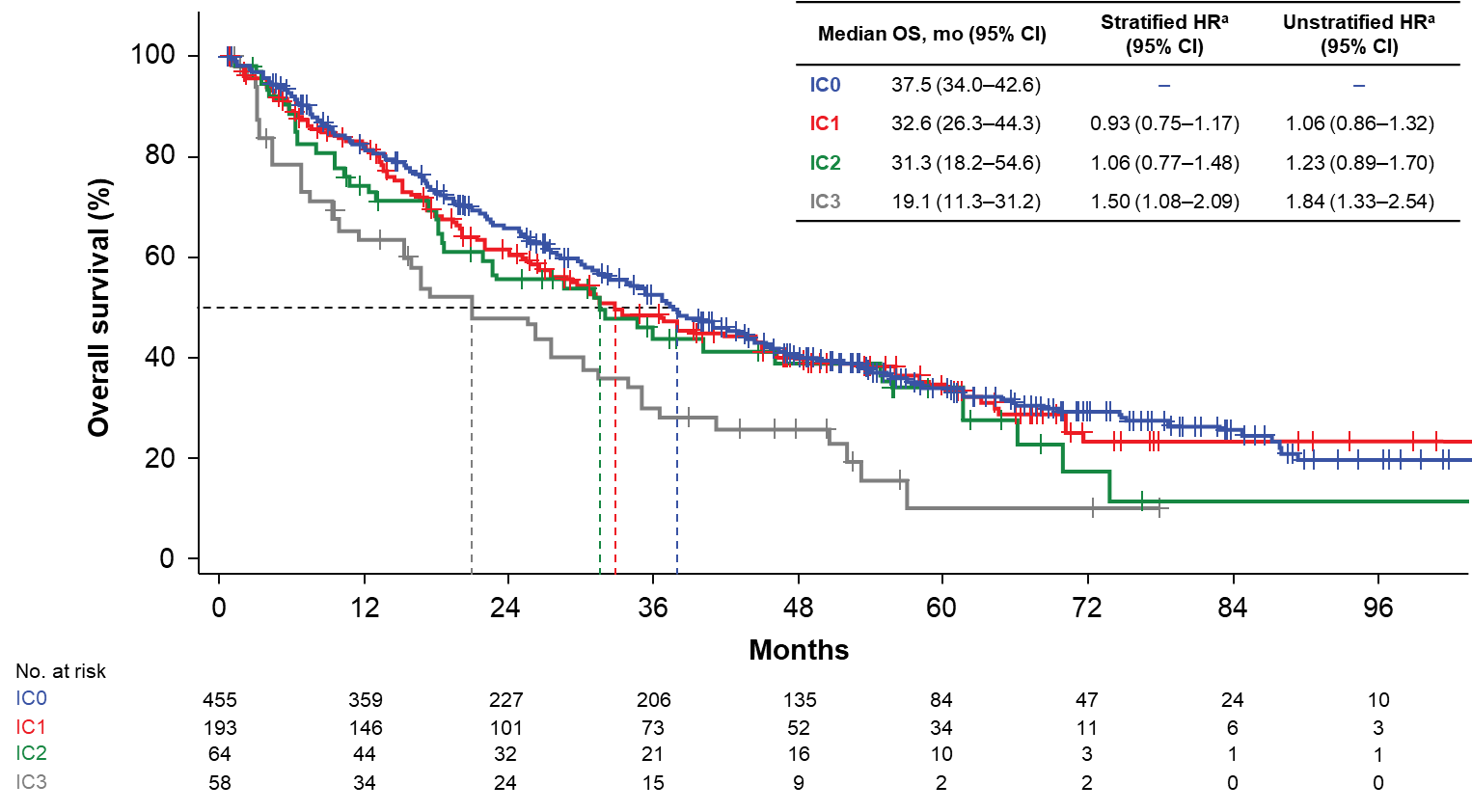
**

**Fig. S8** Analyses in patients who never received CPI. (a) Histograms of pre-/post-weighted propensity score distributions in PD-L1–negative and positive groups in patients who never received CPI. KM curves of (b) OS and (c) PSW-adjusted OS by PD-L1 status in patients who never received CPI. ^a^ Propensity scores were calculated using the same covariates as the primary analysis. CI, confidence interval; CPI, checkpoint inhibitor; HR, hazard ratio; KM, Kaplan-Meier; mo, months; OS, overall survival; PD-L1, programmed death-ligand 1**;** PSW, propensity score-weighted

**a
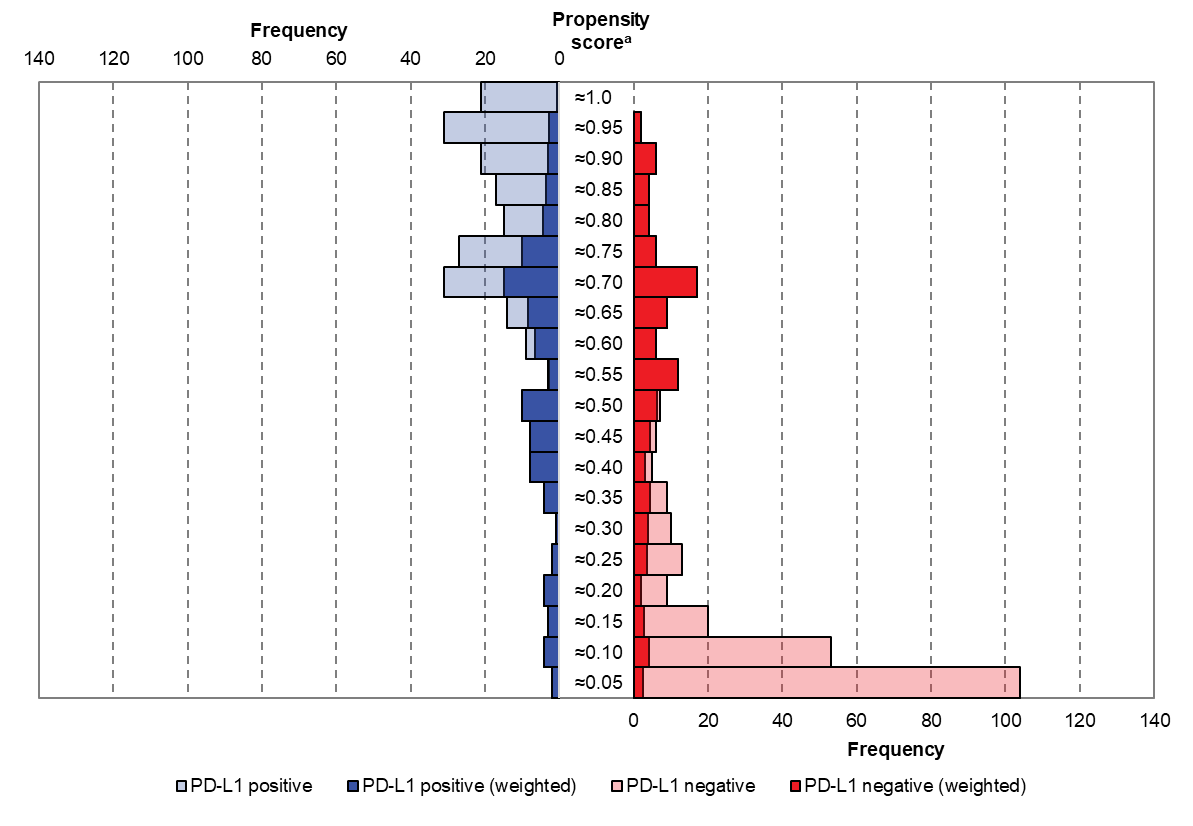
**

**b**

**
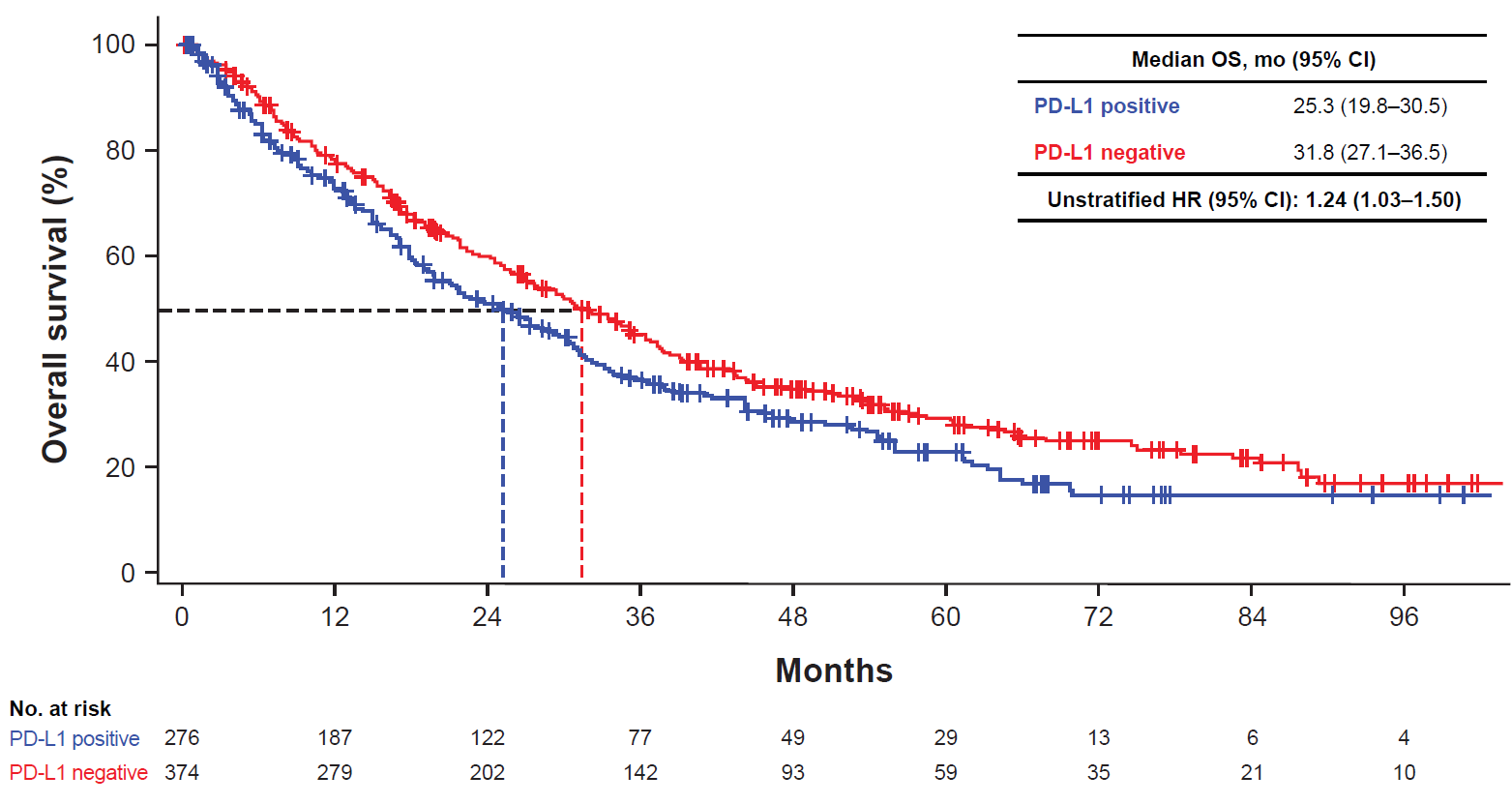
**

**c**

**
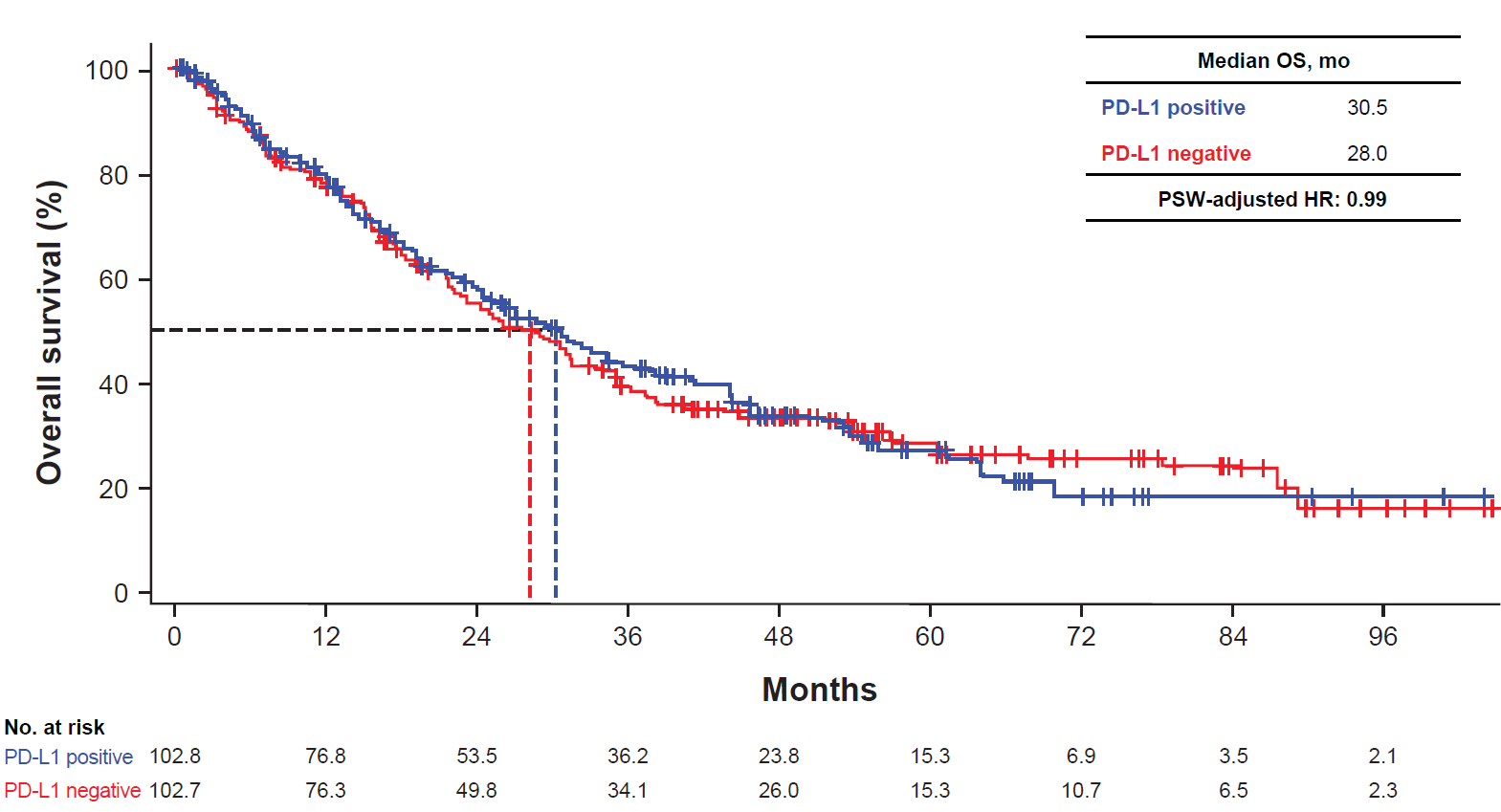
**

**Fig. S9** KM curves of treatment duration for (a) 1L and (b) 2L therapy by PD-L1 status. 1L, first line; 2L, second line; CI, confidence interval; HR, hazard ratio; KM, Kaplan-Meier; PD-L1, programmed death-ligand 1

**a**

**
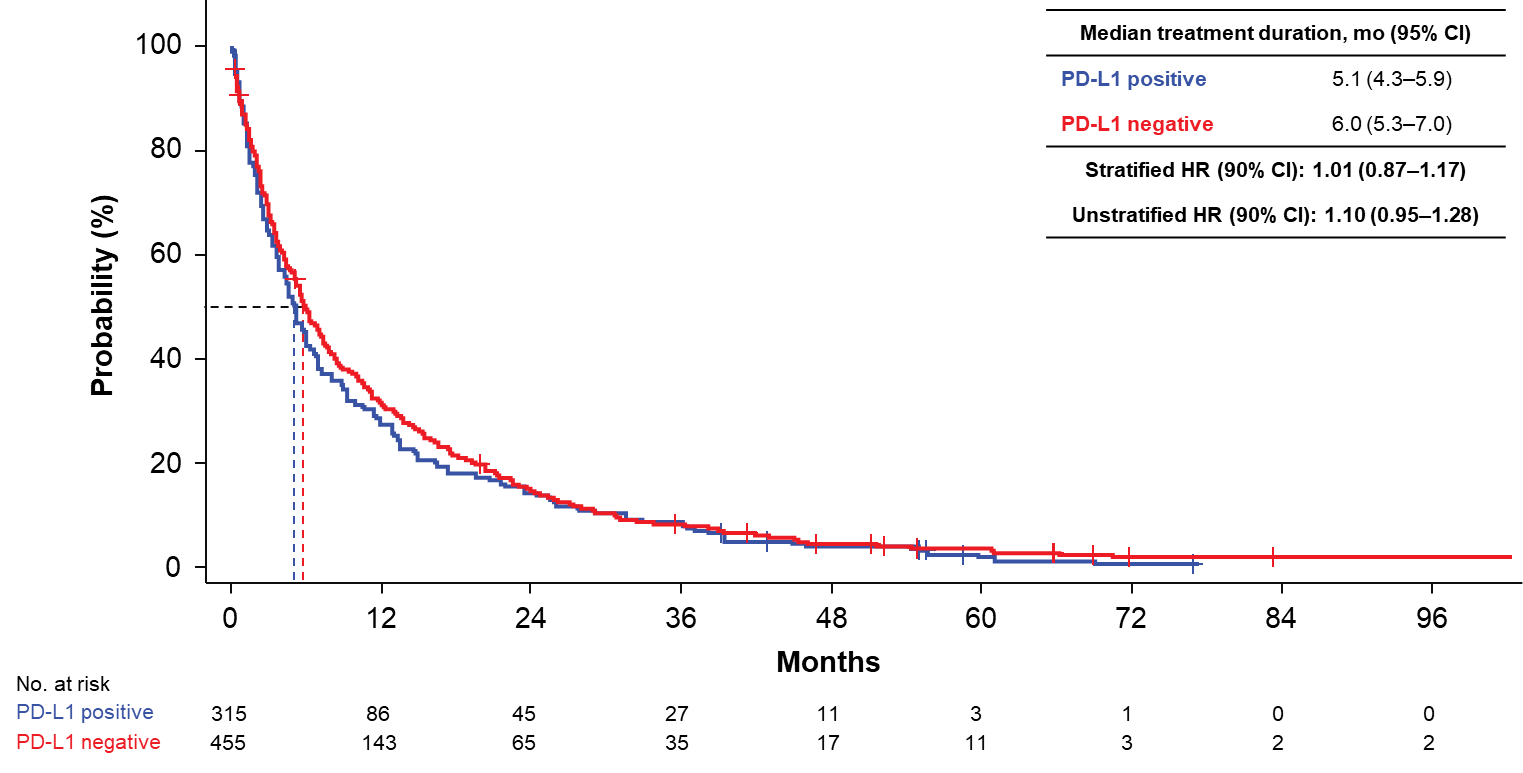
**

**b**

**
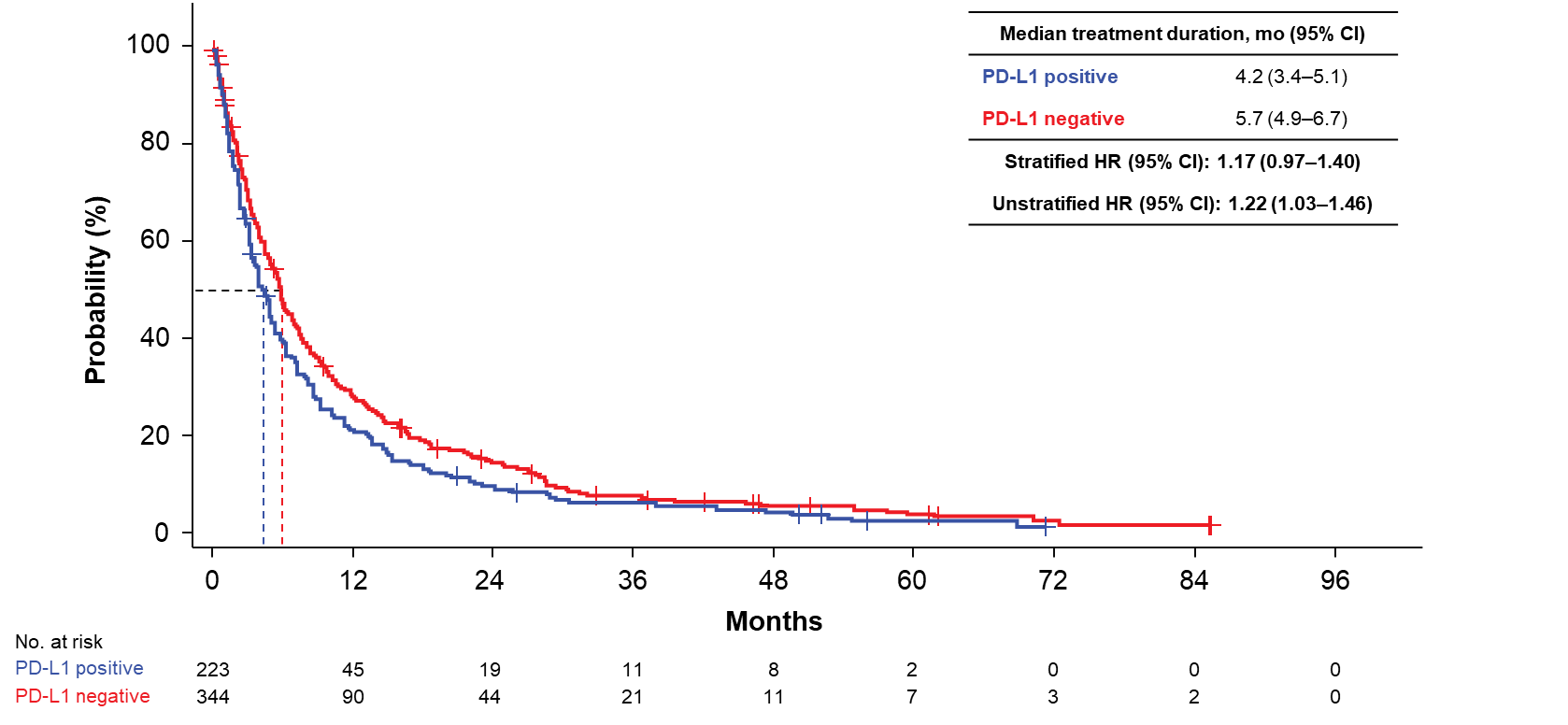
**

**Supplementary Tables**

**Table S1** List of participating sites and investigators

| **Site** | **Principal Investigator** |
| --- | --- |
| Hokkaido University Hospital | T. Osawa |
| Sapporo Medical University Hospital | N. Masumori |
| Hirosaki University Hospital | S. Hatakeyama |
| Iwate Medical University | W. Obara |
| University of Tsukuba Hospital | T. Kojima |
| Toho University Sakura Medical Center | N. Kamiya |
| Keio University Hospital | R. Mizuno |
| Tokyo Medical and Dental University | Y. Fujii |
| Nippon Medical School Hospital | G. Kimura |
| Kitasato University School of Medicine | T. Fujita |
| Yokohama City University Hospital | N. Nakaigawa |
| Niigata University Medical and Dental Hospital | Y. Tomita |
| University of Toyama, University Hospital | Y. Ikehata |
| Shizuoka Cancer Center | R. Yamashita |
| Hamamatsu University School of Medicine | H. Miyake |
| Aichi University Medical Hospital | T. Tsuzuki |
| Nagoya University Hospital | N. Sassa |
| Kyoto University Graduate School of Medicine | O. Ogawa |
| Osaka City University Hospital | S. Tamada |
| Osaka University Hospital | M. Uemura |
| Kindai University Hospital | M. Nozawa |
| Kobe University Hospital | K. Harada |
| Nara Medical University Hospital | S. Anai |
| Okayama University Hospital | A. Takamoto |
| Tokushima University Hospital | T. Fukawa |
| Kagawa University | M. Sugimoto |
| Kyushu University Hospital | K. Tatsugami |
| Nagasaki University Hospital | H. Sakai |
| Kumamoto University Hospital | T. Motoshima |

**Table S2** Logistic regression for propensity score (multivariable)

| **Category** | **Multivariable (N = 637)** | | | | | | |
| --- | --- | --- | --- | --- | --- | --- | --- |
|  | **n** | **Parameter estimate** | **Standard error** | **Odds ratio** | | **Type 3** | |
|  |  |  |  | **Odds ratio** | **95% CI** | **Wald Χ^2^** | ***P* value** |
| Age (y) | 637 | 0.02 | 0.01 | 1.02 | 1.00–1.05 | 3.68 | 0.06 |
| Time from diagnosis to 1L therapy (mo) | 637 | −0.01 | 0.004 | 0.99 | 0.98–1.00 | 3.72 | 0.05 |
| Corrected calcium (mg/dL) | 637 | 0.12 | 0.16 | 1.13 | 0.83–1.56 | 0.60 | 0.44 |
| LDH (IU/L) | 637 | 0 | 0.001 | 1.00 | 1.00–1.00 | 0.0002 | 0.99 |
| Platelet count (/μL) | 637 | 0.01 | 0.02 | 1.01 | 0.98–1.04 | 0.68 | 0.41 |
| Neutrophil count (×10^4^/μL) | 637 | 0.02 | 0.02 | 1.02 | 0.99–1.06 | 1.21 | 0.27 |
| Neutrophil to lymphocyte ratio | 637 | −0.09 | 0.06 | 0.92 | 0.81–1.04 | 1.89 | 0.17 |
| CRP (mg/dL) | 637 | 0.04 | 0.04 | 1.04 | 0.96–1.13 | 0.81 | 0.37 |
| Sex | | | | | | | |
| Male | 486 | – | – | – | – | 0.22 | 0.64 |
| Female | 151 | −0.13 | 0.27 | 0.88 | 0.51–1.50 |  |  |
| Stage at initial diagnosis | | | | | | | |
| I/II | 123 | – | – | – | – | 1.35 | 0.51 |
| III/IV | 497 | 0.39 | 0.34 | 1.47 | 0.76–2.84 |  |  |
| Unknown | 17 | 0.19 | 0.77 | 1.21 | 0.27–5.53 |  |  |
| ECOG PS | | | | | | | |
| 0 | 332 | – | – | – | – | 4.04 | 0.40 |
| 1 | 189 | −0.20 | 0.26 | 0.82 | 0.49–1.37 |  |  |
| 2 | 54 | −0.33 | 0.48 | 0.72 | 0.28–1.83 |  |  |
| 3 or 4 | 13 | −0.48 | 0.85 | 0.62 | 0.12–3.24 |  |  |
| Unknown | 49 | −0.87 | 0.45 | 0.42 | 0.17–1.02 |  |  |
| Anemia | | | | | | | |
| Absent | 292 | – | – | – | – | 0.04 | 0.84 |
| Present | 345 | −0.06 | 0.28 | 0.95 | 0.55–1.64 |  |  |
| MSKCC risk criteria | | | | | | | |
| Favorable risk | 134 | – | – | – | – | 8.30 | 0.02 |
| Intermediate risk | 430 | 1.43 | 0.96 | 4.20 | 0.64–27.44 |  |  |
| Poor risk | 73 | 2.77 | 1.13 | 15.93 | 1.74–145.61 |  |  |
| IMDC risk criteria | | | | | | | |
| Favorable risk | 119 | – | – | – | – | 1.27 | 0.53 |
| Intermediate risk | 380 | −0.60 | 0.97 | 0.55 | 0.08–3.67 |  |  |
| Poor risk | 138 | −0.99 | 1.07 | 0.37 | 0.05–3.03 |  |  |
| Liver metastases at 1L treatment | | | | | | | |
| Absent | 563 | – | – | – | – | 1.96 | 0.16 |
| Present | 74 | −0.52 | 0.37 | 0.60 | 0.29–1.23 |  |  |
| Immune phenotype | | | | | | | |
| Excluded | 320 | – | – | – | – | 132.52 | <0.0001 |
| Inflamed | 43 | 1.35 | 0.55 | 3.85 | 1.31–11.33 |  |  |
| Desert | 274 | −3.03 | 0.28 | 0.05 | 0.03–0.08 |  |  |
| Fuhrman grade | | | | | | | |
| Grades 1 or 2 | 194 | – | – | – | – | 10.80 | 0.01 |
| Grade 3 | 319 | 0.91 | 0.29 | 2.49 | 1.42–4.39 |  |  |
| Grade 4 | 121 | 0.38 | 0.46 | 1.46 | 0.60–3.57 |  |  |
| Indeterminable | 3 | −0.01 | 3.62 | 0.99 | <0.001–>999.999 |  |  |
| Necrosis | | | | | | | |
| Present | 279 | – | – | – | – | 1.28 | 0.53 |
| Absent | 357 | −0.28 | 0.25 | 0.75 | 0.46–1.23 |  |  |
| Indeterminable | 1 | −10.78 | 749.52 | <0.001 | <0.001–>999.999 |  |  |
| Lymphovascular invasion | | | | | | | |
| Present | 162 | – | – | – | – | 2.09 | 0.35 |
| Absent | 445 | −0.31 | 0.27 | 0.73 | 0.44–1.23 |  |  |
| Indeterminable | 30 | 0.26 | 0.60 | 1.30 | 0.40–4.23 |  |  |
| Sarcomatoid component | | | | | | | |
| Present | 74 | – | – | – | – | 3.95 | 0.05 |
| Absent | 563 | −1.00 | 0.51 | 0.37 | 0.14–0.99 |  |  |
| Growth pattern | | | | | | | |
| Expansive pattern | 220 | – | – | – | – | 2.27 | 0.32 |
| Infiltrative pattern | 156 | −0.47 | 0.31 | 0.63 | 0.34–1.15 |  |  |
| Indeterminable | 261 | −0.18 | 0.28 | 0.84 | 0.49–1.44 |  |  |
| C-statistic: 0.89 | | | | | | | |

1L, first line; CRP, C-reactive protein; ECOG PS, Eastern Cooperative Oncology Group performance status; IMDC, International Metastatic Renal Cell Carcinoma Database Consortium; LDH, lactic acid dehydrogenase; MSKCC, Memorial Sloan Kettering Cancer Center.

**Table S3** Logistic regression for propensity scores (univariable)

| **Category** | **Univariable (N = 770)** | | | | | | |
| --- | --- | --- | --- | --- | --- | --- | --- |
|  | **n** | **Parameter estimate** | **Standard error** | **Odds ratio** | | **Type 3** | |
|  |  |  |  | **Odds ratio** | **95% CI** | **Wald Χ^2^** | ***P* value** |
| Age, years | 770 | 0.006 | 0.007 | 1.01 | 0.99–1.0 | – | .39 |
| Time from diagnosis to 1L therapy (mo) | 770 | −0.01 | 0.003 | 0.99 | 0.98–0.99 | – | <.0001 |
| Corrected calcium (mg/dL) | 721 | 0.46 | 0.12 | 1.59 | 1.25–2.01 | – | .0001 |
| LDH (IU/L) | 739 | 0 | 0.0004 | 1.0 | 1.0–1.0 | – | .92 |
| Platelet count (/μL) | 757 | 0.03 | 0.01 | 1.03 | 1.01–1.04 | – | .001 |
| Neutrophil count (×10^4^ /μL) | 681 | 0.01 | 0.01 | 1.01 | 1.0–1.0 | – | .05 |
| Neutrophil to lymphocyte ratio | 680 | 0.05 | 0.03 | 1.05 | 0.99–1.11 | – | .08 |
| CRP (mg/dL) | 727 | 0.094 | 0.023 | 1.10 | 1.05–1.15 | – | <.0001 |
| Sex | | | | | | | |
| Male | 591 | – | – | – | – | 1.38 | .24 |
| Female | 179 | 0.20 | 0.17 | 1.22 | 0.87–1.72 |  |  |
| Stage at initial diagnosis |  |  |  |  |  |  |  |
| I/II | 164 | – | – | – | – | 18.39 | .0001 |
| III/IV | 586 | 0.78 | 0.19 | 2.18 | 1.49–3.19 |  |  |
| Unknown | 20 | −0.13 | 0.55 | 0.88 | 0.30–2.57 |  |  |
| ECOG PS | | | | | | | |
| 0 | 391 | – | – | – | – | 9.80 | .04 |
| 1 | 240 | 0.35 | 0.17 | 1.41 | 1.02–1.96 |  |  |
| 2 | 65 | 0.25 | 0.27 | 1.28 | 0.75–2.18 |  |  |
| 3 or 4 | 20 | 0.06 | 0.47 | 1.06 | 0.42–2.65 |  |  |
| Unknown | 54 | −0.59 | 0.33 | 0.56 | 0.29–1.06 |  |  |
| Anemia | | | | | | | |
| Absent | 354 | – | – | – | – | 18.134 | .0001 |
| Present | 403 | 0.63 | 0.15 | 1.88 | 1.40–2.53 |  |  |
| Unknown | 13 | −0.11 | 0.61 | 0.90 | 0.27–2.99 |  |  |
| MSKCC risk criteria | | | | | | | |
| Favorable risk | 171 | – | – | – | – | 43.38 | <.0001 |
| Intermediate risk | 511 | 1.07 | 0.21 | 2.93 | 1.95–4.40 |  |  |
| Poor risk | 88 | 1.83 | 0.29 | 6.25 | 3.55–11.02 |  |  |
| Unknown | 0 | – | – | – | – |  |  |
| IMDC risk criteria | | | | | | | |
| Favorable risk | 153 | – | – | – | – | 38.17 | <.0001 |
| Intermediate risk | 457 | 0.90 | 0.22 | 2.47 | 1.62–3.77 |  |  |
| Poor risk | 160 | 1.56 | 0.25 | 4.74 | 2.89–7.76 |  |  |
| Unknown | 0 | – | – | – | – |  |  |
| Liver metastases at 1L therapy | | | | | | | |
| Absent | 690 | – | – | – | – | 0.80 | .37 |
| Present | 80 | −0.22 | 0.25 | 0.80 | 0.50–1.30 |  |  |
| Unknown | 0 | – | – | – | – |  |  |
| Immune phenotype | | | | | | | |
| Excluded | 378 | – | – | – | – | 191.64 | <.0001 |
| Inflamed | 59 | 1.52 | 0.42 | 4.57 | 2.02–10.34 |  |  |
| Desert | 333 | −2.84 | 0.22 | 0.06 | 0.04–0.09 |  |  |
| Indeterminable | 0 | – | – | – | – |  |  |
| Fuhrman grade | | | | | | | |
| Grades 1 or 2 | 254 | – | – | – | – | 76.57 | <.0001 |
| Grade 3 | 374 | 1.29 | 0.19 | 3.63 | 2.51–5.25 |  |  |
| Grade 4 | 138 | 1.97 | 0.24 | 7.18 | 4.51–11.43 |  |  |
| Indeterminable | 4 | 0.31 | 1.17 | 1.36 | 0.14–13.35 |  |  |
| WHO/ISUP grade | | | | | | | |
| Grade 1 or grade 2 | 301 | – | – | – | – | 79.95 | <.0001 |
| Grade 3 | 284 | 1.11 | 0.18 | 3.03 | 2.12–4.34 |  |  |
| Grade 4 | 181 | 1.81 | 0.21 | 6.09 | 4.05–9.14 |  |  |
| Indeterminable | 4 | 0.15 | 1.16 | 1.16 | 0.12–11.38 |  |  |
| Necrosis | | | | | | | |
| Present | 331 | – | – | – | – | 55.93 | <.0001 |
| Absent | 437 | −1.14 | 0.15 | 0.32 | 0.24–0.43 |  |  |
| Indeterminable | 2 | −13.51 | 533.39 | <0.001 | <0.001–>999.99 |  |  |
| Lymphovascular invasion | | | | | | | |
| Present | 189 | – | – | – | – | 8.00 | .018 |
| Absent | 539 | −0.47 | 0.17 | 0.62 | 0.45–0.87 |  |  |
| Indeterminable | 42 | −0.16 | 0.34 | 0.85 | 0.44–1.67 |  |  |
| Sarcomatoid component | | | | | | | |
| Present | 87 | – | – | – | – | 28.90 | <.0001 |
| Absent | 683 | −1.32 | 0.24 | 0.27 | 0.17–0.43 |  |  |
| Indeterminable | 0 | – | – | – | – |  |  |
| Growth pattern | | | | | | | |
| Expansive pattern | 278 | – | – | – | – | 3.14 | .21 |
| Infiltrative pattern | 183 | 0.33 | 0.19 | 1.39 | 0.95–2.02 |  |  |
| Indeterminable | 309 | 0.06 | 0.17 | 1.06 | 0.76–1.47 |  |  |

1L, first line; CRP, C-reactive protein; ECOG PS, Eastern Cooperative Oncology Group performance status; IMDC, International Metastatic Renal Cell Carcinoma Database Consortium; LDH, lactic acid dehydrogenase; MSKCC, Memorial Sloan Kettering Cancer Center; WHO/ISUP, World Health Organization/International Society of Urologic Pathologists.

**Table S4** Standardized difference to assess balance of covariates after PSW analysis

| **Category** | **Mean (SD)** | | ***d*** |
| --- | --- | --- | --- |
|  | **PD-L1 positive (IC1/2/3)** | **PD-L1 negative (IC0)** |  |
| Age (y) | 64.77 (10.33) | 64.54 (9.94) | 0.02 |
| Time from diagnosis to 1L therapy (mo) | 19.57 (31.02) | 18.65 (33.34) | 0.03 |
| Corrected calcium (mg/dL) | 9.32 (0.71) | 9.32 (0.80) | 0.01 |
| LDH (IU/L) | 192.77 (109.20) | 196.21 (188.12) | 0.02 |
| Platelet count (/uL) | 24.95 (8.39) | 25.18 (8.98) | 0.03 |
| Neutrophil count (×10^4^/μL) | 64.94 (10.30) | 65.31 (10.12) | 0.04 |
| Neutrophil to lymphocyte ratio | 3.49 (2.83) | 3.61 (3.34) | 0.04 |
| CRP (mg/dL) | 1.63 (3.23) | 1.71 (3.03) | 0.02 |
| Sex | | | |
| Male | 0.77 (0.42) | 0.77 (0.42) | 0.004 |
| Female | 0.23 (0.42) | 0.23 (0.42) | 0.004 |
| Stage at initial diagnosis | | | |
| I/II | 0.18 (0.38) | 0.17(0.38) | 0.01 |
| III/IV | 0.79 (0.41) | 0.80 (0.40) | 0.03 |
| Unknown | 0.03 (0.17) | 0.02 (0.15) | 0.03 |
| ECOG PS | | | |
| 0 | 0.51 (0.50) | 0.48 (0.50) | 0.05 |
| 1 | 0.32 (0.47) | 0.31 (0.46) | 0.009 |
| 2 | 0.08 (0.27) | 0.09 (0.28) | 0.02 |
| 3 | 0.03 (0.16) | 0.03 (0.17) | 0.02 |
| 4 | – | 0.003 (0.053) | – |
| Unknown | 0.07 (0.25) | 0.09 (0.28) | 0.07 |
| Anemia | | | |
| Absent | 0.41 (0.49) | 0.41 (0.49) | 0.01 |
| Present | 0.59 (0.49) | 0.59 (0.49) | 0.01 |
| Unknown | – | – | – |
| MSKCC risk criteria | | | |
| Favorable risk | 0.16 (0.37) | 0.14 (0.35) | 0.05 |
| Intermediate risk | 0.73 (0.44) | 0.74 (0.44) | 0.02 |
| Poor risk | 0.11 (0.31) | 0.12 (0.32) | 0.02 |
| Unknown | – | – | – |
| IMDC risk criteria | | | |
| Favorable risk | 0.15 (0.36) | 0.13 (0.34) | 0.05 |
| Intermediate risk | 0.63 (0.48) | 0.63 (0.48) | 0.01 |
| Poor risk | 0.22 (0.41) | 0.24 (0.43) | 0.05 |
| Unknown | – | – | – |
| Liver metastases at 1L therapy | | | |
| Absent | 0.88 (0.32) | 0.90 (0.30) | 0.05 |
| Present | 0.11 (0.31) | 0.10 (0.30) | 0.02 |
| Unknown | 0.008 (0.091) | – | – |
| Immune phenotype | | | |
| Excluded | 0.76 (0.43) | 0.75 (0.43) | 0.02 |
| Inflamed | 0.04 (0.19) | 0.04 (0.20) | 0.02 |
| Desert | 0.20 (0.40) | 0.21 (0.40) | 0.01 |
| Indeterminable | – | – | – |
| Fuhrman grade | | | |
| Grade 1 | – | 0.008 (0.09) | – |
| Grade 2 | 0.24 (0.43) | 0.24 (0.43) | 0.01 |
| Grade 3 | 0.57 (0.50) | 0.56 (0.50) | 0.02 |
| Grade 4 | 0.19 (0.39) | 0.19 (0.40) | 0.02 |
| Indeterminable | 0.0004 (0.0189) | 0.0003 (0.0186) | 0.001 |
| WHO/ISUP grade | | | |
| Grade 1 | – | 0.008 (0.09) | – |
| Grade 2 | 0.32 (0.47) | 0.33 (0.47) | 0.03 |
| Grade 3 | 0.42 (0.49) | 0.41 (0.49) | 0.03 |
| Grade 4 | 0.26 (0.44) | 0.25 (0.44) | 0.01 |
| Indeterminable | 0.0004 (0.0189) | 0.0003 (0.0186) | 0.001 |
| Necrosis | | | |
| Absent | 0.46 (0.50) | 0.46 (0.50) | 0.01 |
| Present | 0.54 (0.50) | 0.54 (0.50) | 0.01 |
| Indeterminable | – | 0.0000 (0.0001) | – |
| Vascular invasion | | | |
| Absent | 0.25 (0.43) | 0.25 (0.43) | 0.004 |
| Present | 0.71 (0.45) | 0.71 (0.45) | 0.002 |
| Indeterminable | 0.04 (0.20) | 0.04 (0.20) | 0.004 |
| Sarcomatoid component | | | |
| Absent | 0.09 (0.28) | 0.09 (0.29) | 0.01 |
| Present | 0.91 (0.28) | 0.91 (0.29) | 0.01 |
| Indeterminable | – | – | – |
| Growth pattern | | | |
| Expansive pattern | 0.34 (0.48) | 0.34 (0.47) | 0.02 |
| Infiltrative pattern | 0.24 (0.43) | 0.25 (0.43) | 0.01 |
| Indeterminable | 0.41 (0.49) | 0.42 (0.49) | 0.01 |

1L, first line; CRP, C-reactive protein; ECOG PS, Eastern Cooperative Oncology Group performance status; IC0/1/2/3, programmed death-ligand 1 expression level on tumor-infiltrating immune cells; IMDC, International Metastatic Renal Cell Carcinoma Database Consortium; LDH, lactic acid dehydrogenase; MSKCC, Memorial Sloan Kettering Cancer Center; PD-L1, programmed death-ligand 1; PSW, propensity score-weighted; WHO/ISUP, World Health Organization/International Society of Urologic Pathologists.

**Table S5** Propensity score strata per quartile

| **Category** | **<Q1** | | | | **≥Q1 but <Q2** | | | | | **≥Q2 but <Q3** | | | | **Q3 ≤** | | | | | | |
| --- | --- | --- | --- | --- | --- | --- | --- | --- | --- | --- | --- | --- | --- | --- | --- | --- | --- | --- | --- | --- |
|  | **PD-L1**  **positive (IC1/2/3)** | | **PD-L1**  **negative (IC0)** | | **PD-L1**  **positive (IC1/2/3)** | | **PD-L1**  **negative (IC0)** | | | **PD-L1**  **positive (IC1/2/3)** | | **PD-L1**  **negative (IC0)** | | **PD-L1**  **positive (IC1/2/3)** | | | | **PD-L1**  **negative (IC0)** | | |
|  | **n** | **Mean  (SD)** | **n** | **Mean  (SD)** | **n** | **Mean  (SD)** | **n** | **Mean  (SD)** | **n** | | **Mean  (SD)** | **n** | **Mean  (SD)** | | **n** | **Mean  (SD)** | **n** | | **Mean  (SD)** |  |
| Age (y) | 5 | 60.40  (11.28) | 154 | 63.48  (12.07) | 30 | 65.10  (10.28) | 129 | 65.98  (11.36) | 96 | | 64.48  (10.40) | 63 | 63.44  (10.05) | | 138 | 66.48  (10.42) | 22 | | 65.14  (6.62) |  |
| Time from diagnosis to 1L therapy (mo) | 5 | 13.45  (15.84) | 154 | 38.73  (41.81) | 30 | 24.95  (44.60) | 129 | 26.86  (44.55) | 96 | | 18.59  (26.46) | 63 | 15.92  (25.01) | | 138 | 7.26  (14.94) | 22 | | 8.67  (16.71) |  |
| Corrected calcium (mg/dL) | 5 | 9.07  (0.74) | 154 | 9.17  (0.49) | 30 | 9.43  (0.72) | 129 | 9.27  (0.73) | 96 | | 9.18  (0.64) | 63 | 9.28  (0.89) | | 138 | 9.73  (0.89) | 22 | | 9.54  (0.54) |  |
| LDH (IU/L) | 5 | 216.97  (143.37) | 154 | 187.32  (122.58) | 30 | 219.56  (110.12) | 129 | 218.14  (260.99) | 96 | | 175.73  (56.93) | 63 | 182.17  (70.30) | | 138 | 209.25  (226.71) | 22 | | 174.59  (75.48) |  |
| Platelet count (/µL) | 5 | 21.68  (8.94) | 154 | 22.36  (8.05) | 30 | 26.46  (8.07) | 129 | 25.46  (9.93) | 96 | | 24.20  (8.45) | 63 | 24.95  (9.03) | | 138 | 28.63  (9.88) | 22 | | 25.55  (7.60) |  |
| Neutrophil count (×10^4^/μL) | 5 | 70.00  (12.70) | 154 | 63.26  (9.49) | 30 | 66.02  (8.54) | 129 | 65.21  (10.39) | 96 | | 63.79  (10.51) | 63 | 66.63  (10.40) | | 138 | 67.43  (10.23) | 22 | | 62.20  (8.73) |  |
| Neutrophil to lymphocyte ratio | 5 | 5.52  (3.82) | 154 | 2.98  (2.10) | 30 | 3.44  (2.13) | 129 | 3.45  (2.66) | 96 | | 3.20  (2.77) | 63 | 3.85  (3.75) | | 138 | 4.01  (2.84) | 22 | | 3.05  (2.00) |  |
| CRP (mg/dL) | 5 | 0.25  (0.19) | 154 | 0.99  (2.34) | 30 | 1.82  (4.00) | 129 | 1.54  (2.87) | 96 | | 1.46  (2.37) | 63 | 1.68  (3.19) | | 138 | 3.63  (5.07) | 22 | | 1.79  (2.68) |  |

| **Category** | **n** | **%** | **n** | **%** | **n** | **%** | **n** | **%** | **n** | **%** | **n** | **%** | **n** | **%** | **n** | **%** |
| --- | --- | --- | --- | --- | --- | --- | --- | --- | --- | --- | --- | --- | --- | --- | --- | --- |
| **Sex** | | | | | | | | | | | | | | | | |
| Male | 4 | 80.0 | 118 | 76.6 | 23 | 76.7 | 101 | 78.3 | 73 | 76.0 | 48 | 76.2 | 102 | 73.9 | 17 | 77.3 |
| Female | 1 | 20.0 | 36 | 23.4 | 7 | 23.3 | 28 | 21.7 | 23 | 24.0 | 15 | 23.8 | 36 | 26.1 | 5 | 22.7 |
| **Stage at initial diagnosis** | | | | | | | | | | | | | | | | |
| I/II | 1 | 20.0 | 53 | 34.4 | 7 | 23.3 | 24 | 18.6 | 13 | 13.5 | 12 | 19.1 | 12 | 8.7 | 1 | 4.6 |
| III/IV | 4 | 80.0 | 93 | 60.4 | 22 | 73.3 | 102 | 79.1 | 80 | 83.3 | 49 | 77.8 | 126 | 91.3 | 21 | 95.5 |
| Unknown | 0 | 0.0 | 8 | 5.2 | 1 | 3.3 | 3 | 2.3 | 3 | 3.1 | 2 | 3.2 | 0 | 0.0 | 0 | 0.0 |
| **ECOG PS** | | | | | | | | | | | | | | | | |
| 0 | 1 | 20.0 | 87 | 56.5 | 15 | 50.0 | 68 | 52.7 | 48 | 50.0 | 25 | 39.7 | 72 | 52.2 | 16 | 72.7 |
| 1 | 0 | 0.0 | 35 | 22.7 | 9 | 30.0 | 39 | 30.2 | 33 | 34.4 | 23 | 36.5 | 47 | 34.1 | 3 | 13.6 |
| 2 | 0 | 0.0 | 10 | 6.5 | 3 | 10.0 | 10 | 7.8 | 8 | 8.3 | 6 | 9.5 | 15 | 10.9 | 2 | 9.1 |
| 3 | 1 | 20.0 | 2 | 1.3 | 1 | 3.3 | 2 | 1.6 | 2 | 2.1 | 2 | 3.2 | 1 | 0.7 | 1 | 4.6 |
| 4 | 0 | 0.0 | 0 | 0.0 | 0 | 0.0 | 1 | 0.8 | 0 | 0.0 | 0 | 0.0 | 0 | 0.0 | 0 | 0.0 |
| Unknown | 3 | 60.0 | 20 | 13.0 | 2 | 6.7 | 9 | 7.0 | 5 | 5.2 | 7 | 11.1 | 3 | 2.2 | 0 | 0.0 |
| **Anemia** | | | | | | | | | | | | | | | | |
| Absent | 0 | 0.0 | 103 | 66.9 | 12 | 40.0 | 59 | 45.7 | 41 | 42.7 | 27 | 42.9 | 45 | 32.6 | 5 | 22.7 |
| Present | 5 | 100.0 | 51 | 33.1 | 18 | 60.0 | 70 | 54.3 | 55 | 57.3 | 36 | 57.1 | 93 | 67.4 | 17 | 77.3 |
| Unknown | 0 | 0.0 | 0 | 0.0 | 0 | 0.0 | 0 | 0.0 | 0 | 0.0 | 0 | 0.0 | 0 | 0.0 | 0 | 0.0 |
| **MSKCC risk criteria** | | | | | | | | | | | | | | | | |
| Favorable risk | 0 | 0.0 | 73 | 47.4 | 5 | 16.7 | 27 | 20.9 | 14 | 14.6 | 6 | 9.5 | 7 | 5.1 | 2 | 9.1 |
| Intermediate risk | 5 | 100.0 | 79 | 51.3 | 18 | 60.0 | 89 | 69.0 | 78 | 81.3 | 54 | 85.7 | 93 | 67.4 | 14 | 63.6 |
| Poor risk | 0 | 0.0 | 2 | 1.3 | 7 | 23.3 | 13 | 10.1 | 4 | 4.2 | 3 | 4.8 | 38 | 27.5 | 6 | 27.3 |
| Unknown | 0 | 0.0 | 0 | 0.0 | 0 | 0.0 | 0 | 0.0 | 0 | 0.0 | 0 | 0.0 | 0 | 0.0 | 0 | 0.0 |
| **IMDC risk criteria** | | | | | | | | | | | | | | | | |
| Favorable risk | 0 | 0.0 | 65 | 42.2 | 4 | 13.3 | 22 | 17.1 | 14 | 14.6 | 6 | 9.5 | 6 | 4.4 | 2 | 9.1 |
| Intermediate risk | 3 | 60.0 | 79 | 51.3 | 19 | 63.3 | 83 | 64.3 | 64 | 66.7 | 40 | 63.5 | 77 | 55.8 | 15 | 68.2 |
| Poor risk | 2 | 40.0 | 10 | 6.5 | 7 | 23.3 | 24 | 18.6 | 18 | 18.8 | 17 | 27.0 | 55 | 39.9 | 5 | 22.7 |
| Unknown | 0 | 0.0 | 0 | 0.0 | 0 | 0.0 | 0 | 0.0 | 0 | 0.0 | 0 | 0.0 | 0 | 0.0 | 0 | 0.0 |
| **Liver metastases at 1L therapy** | | | | | | | | | | | | | | | | |
| Absent | 5 | 100.0 | 130 | 84.4 | 27 | 90.0 | 113 | 87.6 | 82 | 85.4 | 59 | 93.7 | 128 | 92.8 | 19 | 86.4 |
| Present | 0 | 0.0 | 24 | 15.6 | 2 | 6.7 | 16 | 12.4 | 14 | 14.6 | 4 | 6.4 | 10 | 7.3 | 3 | 13.6 |
| Unknown | 0 | 0.0 | 0 | 0.0 | 1 | 3.3 | 0 | 0.0 | 0 | 0.0 | 0 | 0.0 | 0 | 0.0 | 0 | 0.0 |
| **Immune phenotype** | | | | | | | | | | | | | | | | |
| Excluded | 0 | 0.0 | 0 | 0.0 | 10 | 33.3 | 35 | 27.1 | 96 | 100.0 | 59 | 93.7 | 101 | 73.2 | 19 | 86.4 |
| Inflamed | 0 | 0.0 | 0 | 0.0 | 1 | 3.3 | 0 | 0.0 | 0 | 0.0 | 2 | 3.2 | 37 | 26.8 | 3 | 13.6 |
| Desert | 5 | 100.0 | 154 | 100.0 | 19 | 63.3 | 94 | 72.9 | 0 | 0.0 | 2 | 3.2 | 0 | 0.0 | 0 | 0.0 |
| Indeterminable | 0 | 0.0 | 0 | 0.0 | 0 | 0.0 | 0 | 0.0 | 0 | 0.0 | 0 | 0.0 | 0 | 0.0 | 0 | 0.0 |
| **Fuhrman grade** | | | | | | | | | | | | | | | | |
| Grade 1 | 0 | 0.0 | 0 | 0.0 | 0 | 0.0 | 0 | 0.0 | 0 | 0.0 | 1 | 1.6 | 0 | 0.0 | 0 | 0.0 |
| Grade 2 | 3 | 60.0 | 103 | 66.9 | 9 | 30.0 | 32 | 24.8 | 24 | 25.0 | 17 | 27.0 | 4 | 2.9 | 1 | 4.6 |
| Grade 3 | 2 | 40.0 | 41 | 26.6 | 15 | 50.0 | 78 | 60.5 | 59 | 61.5 | 34 | 54.0 | 75 | 54.4 | 15 | 68.2 |
| Grade 4 | 0 | 0.0 | 8 | 5.2 | 6 | 20.0 | 19 | 14.7 | 13 | 13.5 | 11 | 17.5 | 58 | 42.0 | 6 | 27.3 |
| Indeterminable | 0 | 0.0 | 2 | 1.3 | 0 | 0.0 | 0 | 0.0 | 0 | 0.0 | 0 | 0.0 | 1 | 0.7 | 0 | 0.0 |
| **WHO/ISUP grade** | | | | | | | | | | | | | | | | |
| Grade 1 | 0 | 0.0 | 0 | 0.0 | 0 | 0.0 | 0 | 0.0 | 0 | 0.0 | 1 | 1.6 | 0 | 0.0 | 0 | 0.0 |
| Grade 2 | 3 | 60.0 | 113 | 73.4 | 12 | 40.0 | 39 | 30.2 | 30 | 31.3 | 24 | 38.1 | 11 | 8.0 | 4 | 18.2 |
| Grade 3 | 2 | 40.0 | 29 | 18.8 | 12 | 40.0 | 64 | 49.6 | 43 | 44.8 | 24 | 38.1 | 53 | 38.4 | 10 | 45.5 |
| Grade 4 | 0 | 0.0 | 10 | 6.5 | 6 | 20.0 | 26 | 20.2 | 23 | 24.0 | 14 | 22.2 | 73 | 52.9 | 8 | 36.4 |
| Indeterminable | 0 | 0.0 | 2 | 1.3 | 0 | 0.0 | 0 | 0.0 | 0 | 0.0 | 0 | 0.0 | 1 | 0.7 | 0 | 0.0 |
| **Necrosis** | | | | | | | | | | | | | | | | |
| Absent | 1 | 20.0 | 20 | 13.0 | 11 | 36.7 | 62 | 48.1 | 45 | 46.9 | 27 | 42.9 | 101 | 73.2 | 12 | 54.6 |
| Present | 4 | 80.0 | 133 | 86.4 | 19 | 63.3 | 67 | 51.9 | 51 | 53.1 | 36 | 57.1 | 37 | 26.8 | 10 | 45.5 |
| Indeterminable | 0 | 0.0 | 1 | 0.7 | 0 | 0.0 | 0 | 0.0 | 0 | 0.0 | 0 | 0.0 | 0 | 0.0 | 0 | 0.0 |
| **Vascular invasion** | | | | | | | | | | | | | | | | |
| Absent | 1 | 20.0 | 21 | 13.6 | 8 | 26.7 | 42 | 32.6 | 23 | 24.0 | 15 | 23.8 | 47 | 34.1 | 5 | 22.7 |
| Present | 3 | 60.0 | 128 | 83.1 | 21 | 70.0 | 79 | 61.2 | 70 | 72.9 | 48 | 76.2 | 81 | 58.7 | 15 | 68.2 |
| Indeterminable | 1 | 20.0 | 5 | 3.3 | 1 | 3.3 | 8 | 6.2 | 3 | 3.1 | 0 | 0.0 | 10 | 7.3 | 2 | 9.1 |
| **Sarcomatoid component** | | | | | | | | | | | | | | | | |
| Absent | 0 | 0.0 | 2 | 1.3 | 3 | 10.0 | 12 | 9.3 | 4 | 4.2 | 3 | 4.8 | 46 | 33.3 | 4 | 18.2 |
| Present | 5 | 100.0 | 152 | 98.7 | 27 | 90.0 | 117 | 90.7 | 92 | 95.8 | 60 | 95.2 | 92 | 66.7 | 18 | 81.8 |
| Indeterminable | 0 | 0.0 | 0 | 0.0 | 0 | 0.0 | 0 | 0.0 | 0 | 0.0 | 0 | 0.0 | 0 | 0.0 | 0 | 0.0 |
| **Growth pattern** | | | | | | | | | | | | | | | | |
| Expansive pattern | 0 | 0.0 | 63 | 40.9 | 8 | 26.7 | 38 | 29.5 | 37 | 38.5 | 24 | 38.1 | 44 | 31.9 | 6 | 27.3 |
| Infiltrative pattern | 3 | 60.0 | 29 | 18.8 | 6 | 20.0 | 33 | 25.6 | 23 | 24.0 | 16 | 25.4 | 42 | 30.4 | 4 | 18.2 |
| Indeterminable | 2 | 40.0 | 62 | 40.3 | 16 | 53.3 | 58 | 45.0 | 36 | 37.5 | 23 | 36.5 | 52 | 37.7 | 12 | 54.6 |

1L, first line; CRP, C-reactive protein; ECOG PS, Eastern Cooperative Oncology Group performance status; IC0/1/2/3, programmed death-ligand 1 expression level on tumor-infiltrating immune cells; IMDC, International Metastatic Renal Cell Carcinoma Database Consortium; LDH, lactic acid dehydrogenase; MSKCC, Memorial Sloan Kettering Cancer Center; PD-L1, programmed death-ligand 1; Q, quartile; WHO/ISUP, World Health Organization/International Society of Urologic Pathologists.

**Table S6** Sample collection year and stage at diagnosis

|  | **Full analysis set** | | | **Total**  **(N = 770)** |
| --- | --- | --- | --- | --- |
|  | **Stage at diagnosis** | | |  |
|  | **I/II**  **(n = 164)** | **III/IV**  **(n = 586)** | **Unknown**  **(n = 20)** |  |
| Sample collection year, n (%) | | | | |
| Before 2009 | 84 (10.9) | 95 (12.3) | 14 (1.8) | 193 |
| 2010–2012 | 59 (7.7) | 275 (35.7) | 6 (0.8) | 340 |
| 2013–2015 | 21 (2.7) | 214 (27.8) | 0 (0.0) | 235 |
| After 2016 | 0 (0.0) | 2 (0.3) | 0 (0.0) | 2 |

**Supplementary methods**

**Patient selection**

To avoid bias due to physician selection of specimens for central laboratory evaluation, all patients who met the eligibility criteria during the enrollment period were recruited and programmed death-ligand 1 (PD-L1) status was blinded to the sponsor until the database was locked. The study sites selected were uniformly spread across Japan to ensure that the results obtained could be generalized to the Japanese population. In addition, the sites chosen were those that met pre-specified criteria for the following: average annual number of surgical specimens and number of specimens stored, annual number of patients receiving therapy for recurrent/metastatic renal cell carcinoma (RCC), and investigator choice of standard therapy based on Japanese guidelines.

**Clinical information**

All clinical data were collected via the electronic data system in this study and included information on background, RCC diagnosis, RCC immune phenotype, RCC surgery, systemic therapy for perioperative RCC treatment, systemic therapy for recurrent/metastatic RCC, status at the start of 1L therapy, and dialysis.

**Pathology and immunohistochemistry**

Representative FFPE samples were selected by pathologists in each institution and evaluated by a central pathologist (Tsuzuki T), specialized in genitourinary pathology. Block selection guidance for slides included high-nuclear grade, presence of necrosis, and high immune score (desert < excluded < inflamed).

PD-L1 expression was evaluated independently by two central pathologists (Tsuzuki T and Ohe C) by immunohistochemistry using the VENTANA SP142 assay (Ventana Medical Systems, Inc., 740-4859). Discordant cases were re-evaluated by both pathologists and scored after they reached consensus.

Immune phenotype assessment was performed using CD8 immunostaining by Tsuzuki T.
